# Dynamic Covalent Organocatalysts Discovered from Catalytic Systems through Rapid Deconvolution Screening

**DOI:** 10.1002/chem.201502088

**Published:** 2015-07-14

**Authors:** Fredrik Schaufelberger, Olof Ramström

**Affiliations:** [a] Department of Chemistry, KTH - Royal Institute of Technology Teknikringen 30, 10044 Stockholm (Sweden) E-mail: ramstrom@kth.se

**Keywords:** catalyst screening, dynamic covalent chemistry, organocatalysis, Schiff bases, transimination

## Abstract

The first example of a bifunctional organocatalyst assembled through dynamic covalent chemistry (DCC) is described. The catalyst is based on reversible imine chemistry and can catalyze the Morita–Baylis–Hillman (MBH) reaction of enones with aldehydes or *N*-tosyl imines. Furthermore, these dynamic catalysts were shown to be optimizable through a systemic screening approach, in which large mixtures of catalyst structures were generated, and the optimal catalyst could be directly identified by using dynamic deconvolution. This strategy allowed one-pot synthesis and in situ evaluation of several potential catalysts without the need to separate, characterize, and purify each individual structure. The systems were furthermore shown to catalyze and re-equilibrate their own formation through a previously unknown thiourea-catalyzed transimination process.

## Introduction

Synthesis of new catalysts is critical for modern synthetic chemistry, but catalyst discovery is commonly based on time-consuming and frustrating trial-and-error protocols. To address this issue, many combinatorial approaches to accelerate the process have been developed.[1, 2} However, combinatorial catalysis has been hampered by limited access to structurally diverse systems, in particular with bifunctional scaffolds. Non-trivial synthetic operations are commonly required for their assembly, which renders the systems unsuitable for automated high-throughput synthesis. Furthermore, a significant drawback of most combinatorial catalytic protocols is the requirement for all candidates to be purified, characterized, and evaluated individually, regardless of their activity. Therefore, collective catalyst screening is highly desirable, although only a few pioneering reports have been described.[3]

In recent years, substantial effort has been invested into the design of modular and responsive catalysts, in which the activity can be controlled through secondary inputs. In particular, highly successful self-assembled supramolecular catalysts with tunable activity have been developed for transition-metal catalysis[4] and organocatalysis,[5] providing quick and facile routes to bifunctional catalyst scaffolds. Elegant studies by the Reek[6] and Breit groups,[7] have also shown the potential for simplified screening of such systems by deconvolution methods. However, supramolecular assemblies lack the robustness of covalent linkages. Dynamic covalent chemistry (DCC) uses reversible covalent bonds to mimic the adaptive nature of supramolecular systems, while retaining the advantages of well-defined, stable covalent compounds.[8] For example, DCC has been successfully used for ligand/receptor identification,[9] molecular-interaction analysis,[10] kinetic processes,[11] biopolymers,[12] and chemical reaction networks.[13]

Due to the high interest in developing tunable catalysts and catalytic systems, we became interested in the possibility of creating such a „dynamic“ catalyst and investigating its properties. There are furthermore no known bifunctional catalysts, in which the two functional parts are connected by a reversible covalent bond. The application of DCC for catalyst discovery has otherwise been a long-standing goal.[14] Early examples relied on adaptive host systems that re-equilibrate in the presence of a transition-state analogue (TSA), leading to amplification of the host that in theory best stabilizes the transition state.[15] However, this leads to a need for design and synthesis of the TSA, and the screening process may result in a host that only binds the TSA without possessing any actual catalytic activity.

Because dynamic covalent chemistry is equipped with a developed framework for analysis of large mixtures, we imagined a possibility to directly find an optimal dynamic catalyst for a given reaction from a large adaptive system. Herein, we have developed a method for the dynamic combinatorial synthesis of systems of bifunctional catalysts, followed by in situ identification of the optimal catalyst. The methodology was applied to the challenging Morita–Baylis–Hillman (MBH) reaction, and a selective bifunctional catalyst with interesting properties was discovered.[16] This method circumvents previous issues with DCC and catalysis by directly screening towards the actual chemical transformation in a kinetic manner.

## Results and Discussion

**Figure 1:**
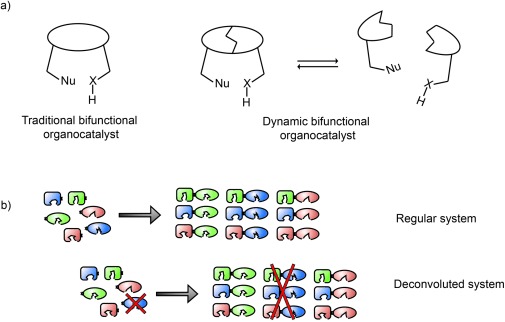
a) Principle of dynamic bifunctional organocatalysis. b) Removal of a single-system component gives propagating effects, eliminating all possible linear combinations of the component.

In bifunctional catalysis, two functional groups capable of activating substrates are mounted on one scaffold.[17] It was hypothesized that if such a scaffold incorporated a reversible bond as shown in Figure 1 a, a dynamic combinatorial system of potential bifunctional catalysts could be generated. By allowing the system to reach equilibrium, a predictable product distribution dictated only by the relative thermodynamic stability of the catalysts would be obtained. Thus, dynamic deconvolution with selective component removal can be used to evaluate the effect of each component (Figure 1 b).[18] Note that the thermodynamic nature of the key bond connection is essential for the accuracy of the deconvolution approach. Performing the same deconvolution on mixtures, in which the bifunctional catalyst has been constructed under kinetic control is not a feasible methodology. Such systems are highly vulnerable to kinetic traps, resulting in a risk of active catalysts being unexpressed in the mixture. For a dynamic system, as long as the building blocks utilized for constructing the catalysts are relatively uniform in terms of the dynamic covalent functional group, all possible linear combinations should be expressed in the system in predictable ratios.

To utilize this DCC methodology for discovery of dynamic bifunctional catalysts, we required a synthetically relevant model transformation. The MBH reaction was chosen, because organocatalysis has proven to be highly successful for this transformation, and the importance of bifunctionality has been well investigated.[19] Furthermore, studies have found that optimal catalyst architectures were difficult to predict through rational design, which together with the often very long reaction times highlighted a need for rapid catalyst screening methods.[19b, e] Traditionally, MBH reactions utilizing α,β-unsaturated ketones as donors are also hard to control, with polymerization and side-reactions often diminishing the efficiency. Accurate catalyst predictions for such a reaction would indicate that the dynamic screening methodology possessed a high level of generality.

**Scheme 1:**
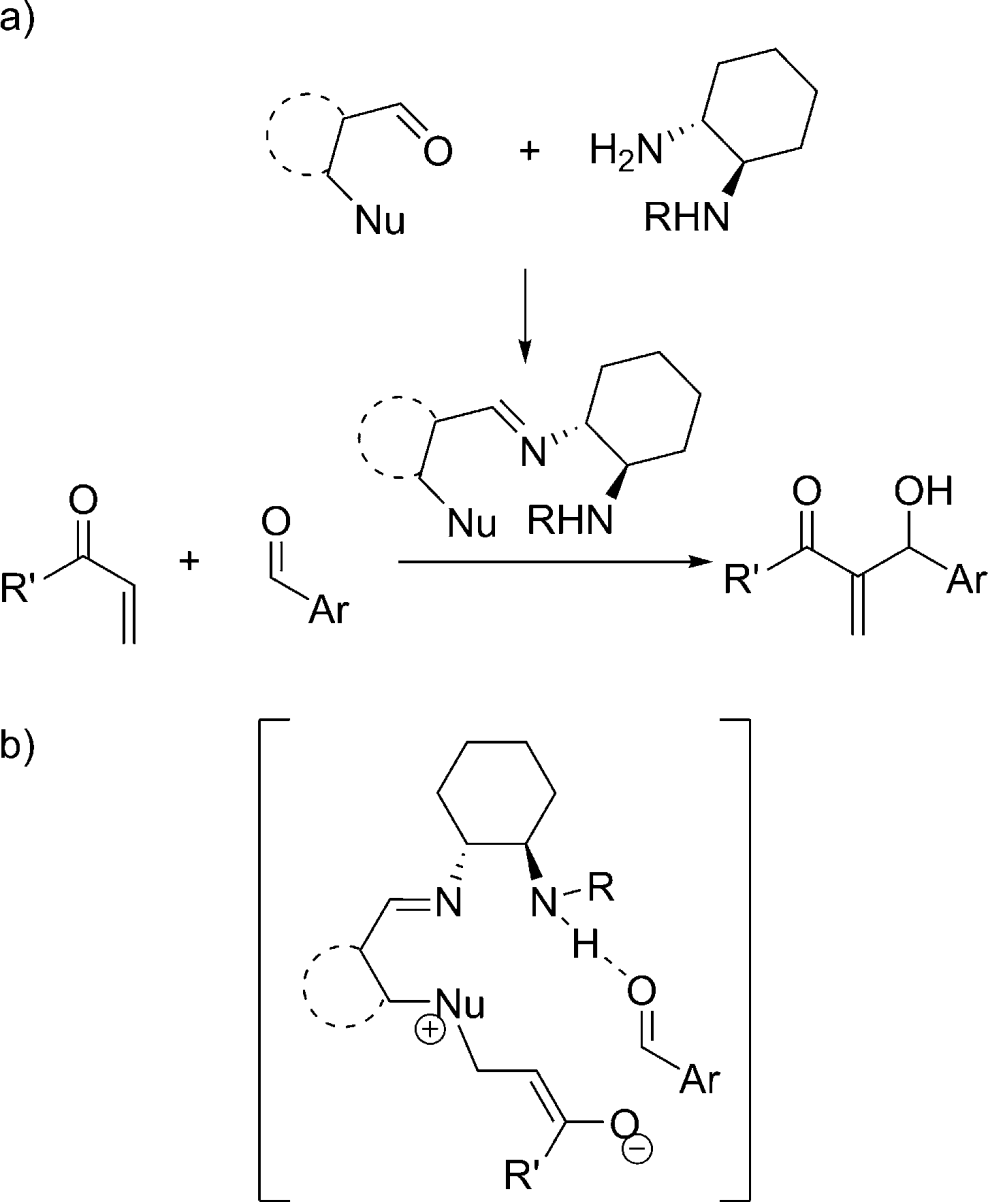
a) Direct bifunctional dynamic catalyst formation for use in an MBH reaction. b) Catalyst preorganizes and activates the substrates towards the MBH reaction.

Thus, a racemic catalyst system that incorporated a nucleophilic Lewis base, an H-bond donor and a dynamic imine bond connecting the two components was designed as shown in Scheme 1 a. Acids and water render the imine bond labile, but removal of either component leads to a structurally robust linkage. This „conditional reversibility“ is essential, because a dynamic catalyst should be able to equilibrate under one set of conditions and stay inert under another. As illustrated in Scheme 1 b, the catalyst should activate both the enone and the aldehyde, and preorganize the substrates for conversion towards the MBH adduct.

**Scheme 2:**
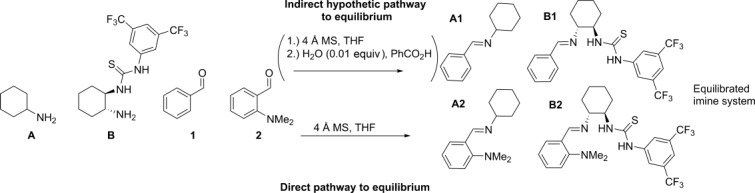
Model-system formation with indirect re-equilibration route (top) and direct condensation route (bottom). THF=tetrahydrofuran, MS=molecular sieves.

The initial strategy was to first form the imines, and then allow the dynamic system to reach equilibrium in situ using an equilibration catalyst. This approach was tested for the model system shown in Scheme 2, using components **A**, **B**, **1**, and **2** to form imines **A1**, **A2**, **B1**, and **B2** quantitatively. Herein, only component **B2** fulfills the criteria for bifunctionality, because it possesses both a nucleophilic tertiary amine moiety and an H-bond donating thiourea group.

However, upon attempted re-equilibration by addition of catalytic amounts of water and widely used transimination catalysts, such as benzoic acid or Sc(OTf)_3_, it was noticed that the component distribution in the imine system did not change. Control experiments confirmed that the system had in fact already reached equilibrium during condensation (see the Supporting Information). This result was surprising, because amines and aldehydes in the absence of acid are known to condensate irreversibly under kinetic control.

It is known that thiourea moieties form strong H bonds with imines. It was thus hypothesized that the thiourea N–H protons could act as general acid catalysts for the system and self-catalyze the system synthesis, in which it takes part. Further control experiments indicated that thioureas are indeed able to induce equilibration of dynamic imine systems, as long as water and/or amines are still present in the mixture (see the Supporting Information). We also confirmed that transimination did not proceed at all in the absence of these species, which supports a hydrolysis/condensation mechanism for the re-equilibration. This effectively led to dynamic systems that were „locked“ at equilibrium under dry conditions, because the water necessary for re-equilibration was continuously removed during the condensation phase. Furthermore, it was also confirmed that thiourea structures were capable of catalyzing the exchange even in the absence of primary amines, indicating that aliphatic amine transimination catalysis was not the sole factor at play. To the best of our knowledge, this is the first report of H-bond-catalyzed transimination outside of biological systems. This finding greatly simplified our method, because the re-equilibration step shown in Scheme 2 could be entirely omitted. Furthermore, it added a further layer of complexity to this potential catalyst class, because these dynamic thiourea-imine catalysts are, in a sense, able to modify and catalyze their own formation.

**Scheme 3:**
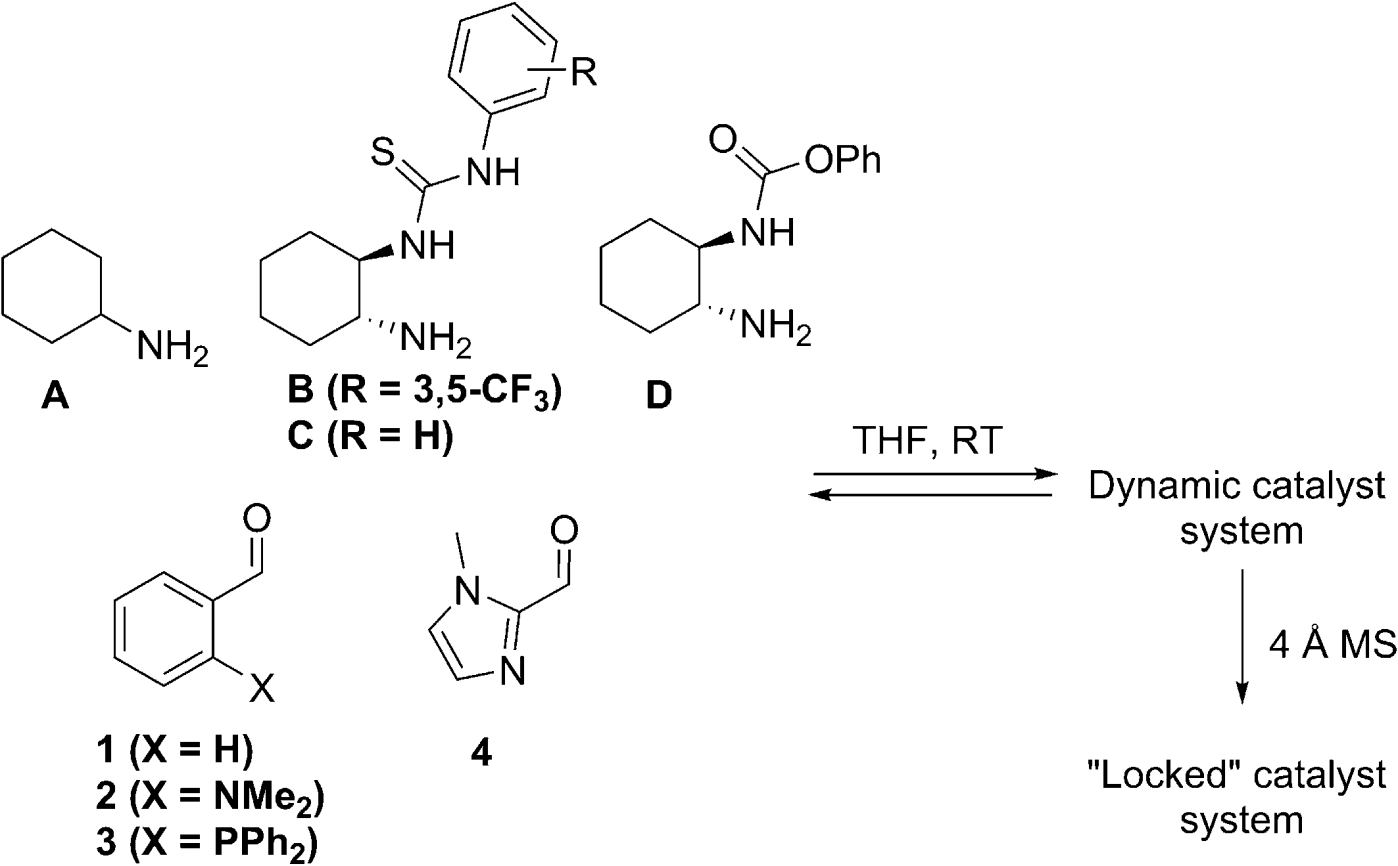
Formation of dynamic 16-component imine system and „locking“ by water removal.

**Figure 2:**
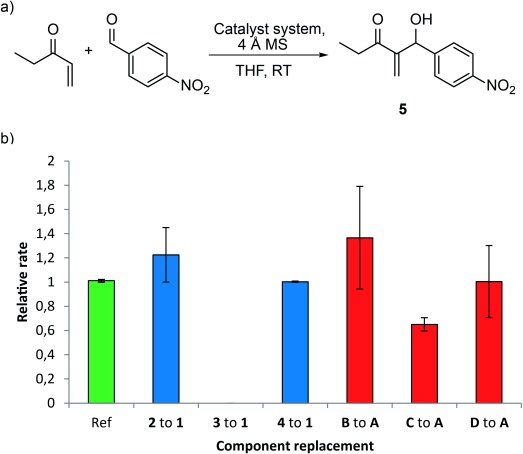
a) Model MBH reaction. b) Observed initial rate difference for MBH reaction upon selective replacement of investigated components 2–4 or B-D by equivalent amount of non-functionalized analogues 1 or A in the pre-generated catalyst system. Conditions: 0.12 mmol *p*-nitrobenzaldehyde, 0.24 mmol ethyl vinyl ketone, 4 Å MS (300 mg), anhydrous THF (0.5 mL), pre-generated imine catalyst system (0.075 mmol of each initial component A-D and 1–4 except for the omitted building block and the replacement compound A or 1 of which 0.15 mmol was added). Duplicate experiments; for further experimental details and kinetic plots, see the Supporting Information.

With equilibration conditions in hand, the system was next expanded to four aldehydes and four amines, as shown in Scheme 3, to increase the chances of finding an active catalyst. Aldehydes **2**, **3**, and **4** comprise nucleophilic sites in the *ortho* position to the imine linker, whereas amines **B**, **C**, and **D** incorporate H-bond donors. Cyclohexylamine **A** and benzaldehyde **1** were used as controls. A dynamic catalyst system composed of 16 different imines was formed analogously to the model reaction, and equilibrium was again attained during the condensation phase. Next, ethyl vinyl ketone and *p*-nitrobenzaldehyde were added directly to the system as shown in Figure 2 a. The MBH reaction proceeded readily, and 20–25 % yield of the desired adduct **5** was obtained after 24 h, as indicated by NMR analysis. Thus, at least one of the 16 potential catalysts in the mixture possessed MBH activity.

To minimize the number of experiments required to identify the active components in the mixture, a dynamic deconvolution scheme was devised, the results of which are shown in Figure 2 b. Equimolar amounts of the amine and aldehyde species were generally required, because the formed imines were inert under MBH conditions even in the presence of thioureas. Hence, deconvolution could be efficiently accomplished through selective replacement of the evaluated component by an equivalent amount of a reference compound (**A** for amines, **1** for aldehydes). Initial rates were then measured to fully correlate systemic catalytic activity with changes in system composition upon component replacement.[20] Replacement of potentially active components by inactive species would lead to retarded rates of the investigated reaction, compared with the complete system with all functionalities present (the reference bar in Figure 2 b). Conversely, removal of a component that is detrimental to catalytic activity should give enhanced initial rates.

As can be seen from Figure 2 b, replacement of the dimethylamino-containing component **2** gave a slight rate increase. A potential explanation for this observation can be the systemic effects of bifunctionality in the catalyst system. Assuming one or more optimal combinations of nucleophile and H-bond donor, a scenario, in which pairing of an inactive component with a potentially active species would produce a bifunctional catalyst that exhibits low activity, can be envisaged. If this pairing would be thermodynamically more preferred than pairing of two active components, then removal of the inactive component would lead to re-equilibration in favor of the more active catalyst combination and thus increased rates. This scenario may be well applicable to the case of component **2**. However, removal of diphenylphosphine-containing aldehyde **3** led to complete loss of catalytic activity, implying that the highly nucleophilic phosphine was the only nucleophile in the system capable of catalyzing the reaction. In further support of this observation, imidazole-based aldehyde **4** showed almost no rate change when replaced.

The results from the H-bond donor screen showed less pronounced differences. Removal of the weaker H-bonding thiourea **C** provided the largest systemic effect, with the product formation rate decreasing by almost 30 %. Replacement of the stronger H-bond donor **B** instead led to a rate increase, suggesting that **B** had deleterious effects on the catalysis.

**Figure 3:**
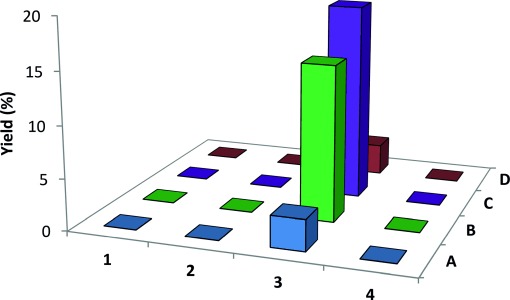
Yields of compound 5 in parallel catalyst-screening experiments. Conditions: 0.1 mmol *p*-nitrobenzaldehyde, 0.3 mmol ethyl vinyl ketone, 0.02 mmol bifunctional catalyst, 0.5 mL THF, 200 mg 4 Å MS, 24 h, RT.

To evaluate the accuracy of the deconvolution predictions, a parallel screening test was subsequently performed. All linear combinations of the catalysts were synthesized in situ by direct condensation of the corresponding amine and aldehyde, and tested in single experiments. Only the four reactions involving the imines resulting from aldehyde **3** showed any product formation after 24 h. These four catalysts were then synthesized and purified, giving bench-stable compounds that were subsequently tested in controlled single experiments. The results are summarized in Figure 3 and are in accordance with the dynamic deconvolution results. Compound **C3** turned out to be the most active catalyst, with a 19 % yield of the MBH product **5**, compared to 15 % for **B3** and only 3 % for **A3** and **D3**.

The relatively high catalytic ability of **B3** was initially surprising, because the system experiments actually predicted the compound to be detrimental to catalysis. However, subsequent experiments showed that **B3** was highly unselective, with formation of large amounts of byproducts. Furthermore, product **5** was shown to be unstable in the presence of **B3**, and decomposed over time. These effects are an example of why care has to be taken in the collective screening of catalyst mixtures, because simple determination of the yield of **5** upon completed reaction would not lead to accurate predictions of the optimal catalyst activities. However, this study has showcased that kinetic measurements of initial rates is a possible way to measure systemic activities of catalyst mixtures.

Although **C3** is by no means a state-of-the-art catalyst activity-wise, these results provide compelling evidence that the deconvolution methodology has accurately predicted the most active catalyst from a dynamic system. This protocol seems to be highly suited for detecting components crucial for activity, but it can also differentiate between less important functional groups that still contribute to the catalysis in the system. The method is simple and straightforward, and allows one-pot synthesis and subsequent screening of well-defined, covalently linked bifunctional organocatalysts without the need for separation, purification, and characterization of each individual molecule. The small model system investigated in this study is easily amenable to expansion, and the deconvolution protocol would be expected to increase further in efficiency with larger systems. Furthermore, considering the range of dynamic covalent linkages developed in recent years, a wide range of potential dynamic catalysts architectures could be envisaged.

**Scheme 4:**
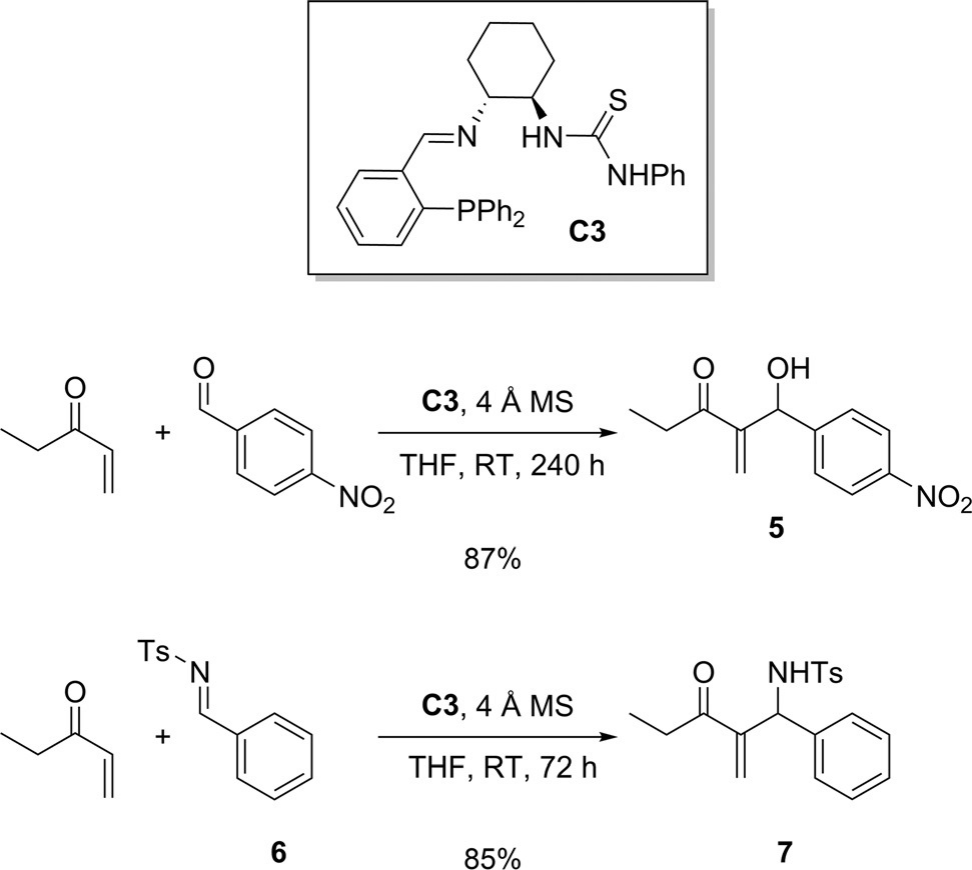
Catalyst performance in MBH and aza-MBH reactions. Conditions: 0.2 mmol aldehyde/imine 6, 0.6 mmol ethyl vinyl ketone, 0.04 mmol C3, 4 Å MS (100 mg), 1.0 mL THF, N_2_.

Having shown that the dynamic covalent chemistry enabled accelerated activity screening, we turned to investigating the behavior of the dynamic bifunctional catalyst **C3** in more detail. When the MBH reaction was performed with 20 % loading of **C3**, a yield of 87 % could be provided after an extended reaction time (240 h). In comparison, a maximum of only 27 % yield could be obtained using **B3**. Also, **C3** could efficiently catalyze an aza-MBH reaction with highly electrophilic phenyl *N*-tosyl imine **6** to give aza-MBH adduct **7** in a very good 85 % yield over 72 h (Scheme 4).[21]

Furthermore, we were interested in investigating if the dynamic covalent bond could be utilized to modulate the MBH activity. Running the reaction with only amine **C** predictably only led to imine formation with *p*-nitrobenzaldehyde, but more surprisingly, utilizing aldehyde **3** as the sole catalyst led to almost no product and quick decomposition (Table 1). When adding **C** and **3** together, the MBH reaction proceeded with very low selectivity and yield, with decomposition of the aldehyde presumably occurring over MBH adduct formation. However, when **C** and **3** were pre-stirred with 4 Å MS overnight, **C3** was formed in quantitative yield, and the corresponding MBH reaction proceeded readily and selectively. Conversely, pre-stirring four equivalents of H_2_O with **C3** followed by reagent addition again produced almost no product formation, because the thiourea seemed to have catalyzed the partial hydrolysis of the imine back to the unfavorable aldehyde–amine pair. These results indicate that the dynamic bifunctional organocatalysts might be utilized as primitive switches, especially given the discovered self-modifying capabilities of this class of catalysts.

**Table 1:** Tunable catalytic activity for C3^[a]^

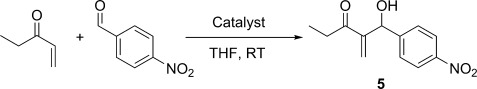		
Catalyst	Incubation time [h]	MBH activity^[b]^
**C3**	–	ON
**C**	–	OFF
**3**	–	OFF^[e]^
**C3**^[c]^	24	OFF
**C**+**3**	–	OFF^[e]^
**C**+**3**^[d]^	24	ON

[a] Conditions: 0.1 mmol *p*-nitrobenzaldehyde, 0.3 mmol ethyl vinyl ketone, 0.02 mmol catalyst, 0.5 mL THF, N_2_. [b] Indicated by ^1^H NMR spectroscopy after 7 h. [c] With 0.2 mmol H_2_O. [d] With 4 Å MS (100 mg). [e] Trace amounts.

The inclusion of a dynamic imine bond, as well as a transimination catalyst, into the same structure also opens further interesting possibilities. For the catalyst screening, the dynamic system was „locked“ during the entire catalytic event to maintain accuracy in reaction kinetics measurements. However, it is also straightforward to „unlock“ the dynamic system and allow living dynamic catalyst behavior, in which the catalyst structure is continuously changing during the reaction. In theory, organocatalysts capable of in situ error correction of their own molecular architecture could then be envisaged.

## Conclusion

A new class of dynamic bifunctional catalysts capable of catalyzing modifications of their own constitution was developed, and it was showcased how this property allows one-pot synthesis and evaluation of large systems of catalysts. The methodology uncovered a relatively effective catalyst for the Morita–Baylis–Hillman reaction, and catalyst effectiveness could be regulated through manipulations of the dynamic covalent bond. DCC is integral for the screening approach, because it enables a deconvolution strategy that rapidly identifies the system components that contribute most to catalytic activity. The dynamic imine linkage allows proofreading of the dynamic system, with the reversibility ensuring a uniform catalyst distribution. The methodology can be utilized for catalyst discovery, and the obtained dynamic bifunctional scaffolds exhibit the potential for use as adaptable organocatalysts. Furthermore, this also marks the first report of thiourea-catalyzed transimination. Further investigations on the screening methodology and the self-modifying ability of the dynamic catalysts are currently in progress.

## Experimental Section

### Experimental procedure for dynamic system generation

Aldehydes and amines (0.075 mmol each) were dissolved in anhydrous THF (0.5 mL) in an Eppendorf vial, and the solution was transferred to a dry reaction vial containing pre-activated 4 Å MS (300 mg) under N_2_. The mixture was stirred at room temperature for 20 h after which time the equilibrated system was obtained. Tests for thiourea system equilibration were performed (see the Supporting Information), showing that the systems were at equilibrium after condensation.

### Kinetic analysis of Morita-Baylis–Hillman reactions with dynamic systems catalysis

A dynamic system was generated according to the description above. Afterwards, *p*-nitrobenzaldehyde (18.1 mg, 0.12 mmol) in anhydrous THF (0.120 mL) was added under N_2_, followed by addition of ethyl vinyl ketone (23.9 μL, 20.8 mg, 0.24 mmol). The mixture was stirred at room temperature under N_2_. An aliquot of the reaction mixture (30.0 μL) was withdrawn and added to 0.550 mL CDCl_3_ in an NMR tube, with PhSiMe_3_ (0.020 μL/mL CDCl_3_) as internal standard. NMR measurements were performed within 5 min, although control experiments indicated that the aliquot composition was stable for several hours in anhydrous CDCl_3_. Product formation was monitored by integrating the characteristic peaks at *δ*=5.66 and 6.00 ppm and comparing to the internal standard.

## Supporting Information

As a service to our authors and readers, this journal provides supporting information supplied by the authors. Such materials are peer reviewed and may be re-organized for online delivery, but are not copy-edited or typeset. Technical support issues arising from supporting information (other than missing files) should be addressed to the authors.

miscellaneous_information
