# Supplementary material for: Dynamic Covalent Organocatalysts Discovered from Catalytic Systems through Rapid Deconvolution Screening
Source: Chemistry. 2015 Jul 14;21(36):12735–40. doi: 10.1002/chem.201502088 (PMC4557047; doi:10.1002/chem.201502088)
Supplement: Supplementary file 1 — miscellaneous_information [file chem0021-12735-sd1.pdf]

CHEMISTRY
A European Journal

Supporting Information

Dynamic Covalent Organocatalysts Discovered from Catalytic Systems through Rapid Deconvolution Screening

Fredrik Schaufelberger and Olof Ramström*[a]

chem_201502088_sm_miscellaneous_information.pdf

Supporting information

Dynamic Covalent Organocatalysts Discovered From Catalytic Systems Through Rapid Deconvolution Screening

*Fredrik Schaufelberger and Olof Ramström**

KTH - Royal Institute of Technology, Department of Chemistry,
Teknikringen 30, S-10044 Stockholm, Sweden; Email: ramstrom@kth.se

CONTENTS

General methods and materials **1**
Synthesis of starting materials **2**
Synthesis and characterization of imine catalysts **5**
General synthetic procedure for imine condensation reactions 5
Experimental procedure for Morita-Baylis-Hillman reactions 7
Investigation of transimination catalysis by thiourea **8**
Model system generation 8
Equilibrium manipulation 8
Thiourea-catalyzed condensation and equilibration 9
H_2O-dependence on thiourea-transimination 10
Further tests for transimination catalysis by thiourea 10
Procedure for system generation and deconvolution experiments **11**
Experimental procedure for system generation 11
Experimental procedure for Morita-Baylis-Hillman reactions with dynamic system catalysis 11
Kinetic analysis of Morita-Baylis-Hillman reactions 12
MBH Catalyst evaluation experiments 12
Initial rate data for MBH system catalysis screening 13
References 15
Spectral data for new compounds 16

GENERAL METHODS AND MATERIALS

All chemicals throughout this work were purchased from commercial suppliers with the highest available purity, and used as received, except benzaldehyde, salicylaldehyde and 2-bromobenzaldehyde which were distilled under anhydrous conditions at reduced pressure prior to use and stored under N_2. The reactions using air or moisture sensitive compounds were carried out with oven-dried glassware under an atmosphere of N_2 or argon as specified in the synthetic descriptions. Molecular sieves 4 Å were pre-activated by heating to 600 °C under reduced pressure for 15 minutes followed by extended storage at 150 °C. All anhydrous solvents were passed through alumina columns in a Glass Contour solvent dispensing system and stored over molecular sieves, with the exception of 1,4-dioxane, which was bought from commercial suppliers and used without further purification, and chloroform, which was dried through fractional distillation (atmospheric pressure) over $CaCl_2$. Deuterated chloroform for NMR analysis was filtrated through a plug of anhydrous K_2CO_3 to remove acidic impurities, followed by drying over activated 4 Å MS under inert atmosphere. Solvents for workup, extractions and flash column chromatography were of analytical grade and used as supplied. Reactions were monitored by NMR spectroscopy or thin layer chromatography using pre-coated Merck silica gel 60 F254 alumina plates (0.25 mm). Visualization was accomplished using UV light (254 nm), followed by staining in a diluted solution of phosphomolybdic acid in ethanol, in $KMnO_4$/NaOH aqueous solution, or 5% H_2SO_4 in ethanol with heating. Flash column chromatography was carried out using Merck silica gel 60 (0.040-0.063

mm). Concentration *in vacuo* was performed at ≈10 mbar and 30-40°C, drying at the high vacuum at ≈ 0.5 torr and room temperature. HRMS was performed at the Institute of Chemistry at University of Tartu, Estonia. ATR-IR spectroscopy was performed on a Thermo Scientific Nicolet iS10 spectrophotometer. A Bruker Avance DMX 500 MHz NMR spectrometer was used for recording ^{1}H-NMR, ^{13}C-NMR and ^{31}P-NMR, while a Bruker Avance 400 spectrometer and a Bruker Ascend 400 spectrometer (both 400 MHz) were used for the kinetic studies and ^{19}F NMR spectra. Chemical shifts are reported as δ values (ppm) relative to tetramethylsilane (Me_4Si) with residual undeuterated $CHCl_3$ (^{1}H NMR δ 7.26, ^{13}C NMR δ 77.16) or DMSO (^{1}H NMR δ 2.50, ^{13}C NMR δ 39.52) as internal standards. Shifts for ^{31}P- and ^{19}F-NMR are reported relative to H_3PO_4 and $CFCl_3$, respectively. All *J* values are given in Hertz (Hz).

SYNTHESIS OF STARTING MATERIALS

1-((±)-2-aminocyclohexyl)-3-(3,5-bis(trifluoromethyl)phenyl)thiourea (B)[1]

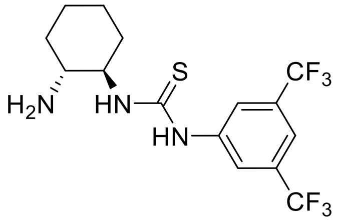

To a solution of (±)-*trans*-diaminocyclohexane (0.582 g, 5.1 mmol) in anhydrous CH_2Cl_2 (10 mL) was added a solution of 3,5-bis(trifluoromethyl)phenyl isothiocyanate (0.93 mL, 1.38 g, 5.1 mmol) in CH_2Cl_2 (10 mL) dropwise over 20 minutes under N_2. After addition was finished, the solution was stirred for 3 h at room temperature, until all starting material was consumed according to TLC. The slightly yellow solution was concentrated in vacuo to yield the crude product as a yellow solid. Further purification by column chromatography (CH_2Cl_2/MeOH/NEt_3 40:1:0.1) yielded the product (1.63 g, 4.2 mmol, 83% yield) as a white powdery solid. **^{1}H NMR** (500 MHz, $CDCl_3$), δ_H = 8.01 (s, 2H), 7.56 (s, 1H), 6.52 (s, br, 1H), 4.16 (s, br, 1H), 3.36 (s, br, 1H), 2.03-1.93 (m, br, 2H), 1.81-1.65 (m, 2H), 1.35-1.18 (m, 4H); **^{13}C NMR** (125 MHz, $CDCl_3$), δ_c = 183.2, 158.0, 131.6 (q, *J* = 33 Hz), 123.1 (q, *J* = 271 Hz), 122.6, 117.4, 63.0, 56.6, 34.9, 32.1, 24.6, 24.5;

1-((±)-2-aminocyclohexyl)-3-phenylthiourea (C)[2]

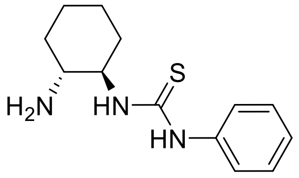

To a solution of (±)-*trans*-diaminocyclohexane (2.14 g, 18.7 mmol) in anhydrous CH_2Cl_2 (250 mL) was added phenyl isothiocyanate (1.50 mL, 1.69 g, 12.5 mmol) dropwise over 20 minutes at 0 °C under N_2. The reaction was allowed to warm to room temperature overnight, and after 17 h the starting material was consumed according to TLC analysis. The yellow solution was concentrated in vacuo to yield the crude product as a yellow foam. Further purification by column chromatography (CH_2Cl_2/MeOH/NEt_3 40:1:0.1→9:1:0.1) yielded the product (2.84 g, 11.4 mmol, 91% yield) as a white powdery solid. **^{1}H NMR** (500 MHz, $CDCl_3$) δ_H = 8.64 (s, br, 1H), 7.39-7.17 (m, 5H), 6.37 (s, 1H), 4.13 (s, br, 1H), 2.47 (s, br, 1H), 2.17-2.08 (m, 1H), 1.95-1.86 (m, 1H), 1.74-1.65 (m, 2H), 1.37-0.95 (m, 4H); **^{13}C NMR** (125 MHz, $CDCl_3$), δ_c = 180.7, 136.6, 129.8, 126.5, 124.7, 61.5, 55.7, 35.5, 32.0, 24.8, 24.7.

(±)-2-((phenoxycarbonyl)amino)cyclohexan-1-aminium iodide and phenyl((±)-2-aminocyclohexyl)carbamate (D•HI)[3]

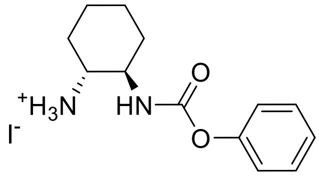

To a solution of (±)-*trans*-diaminocyclohexane (1.713 g, 15 mmol) in isopropanol (15 mL) was added diphenyl carbonate (3.213 g, 15 mmol) and the resulting solution was stirred at reflux under N_2. After 30 minutes, a white precipitate was formed and the solvent was removed in vacuo. To ensure complete isopropanol removal, the residual solid was redispersed in anhydrous toluene and the solvent removed. This process was repeated three times, followed by drying under high vacuum. The obtained white solid was mixed with methanesulfonic acid (0.97 mL, 1.44 g, 15.0 mmol) and the mixture turned homogenous upon heating. After stirring the red solution under argon at 110 °C for 1 h the mixture was allowed to cool to room temperature. Distilled H_2O (15 mL) was slowly added, leading to precipitation. The phenol obtained as a byproduct was removed by extracting with ethyl acetate (3 x 15 mL, complete separation of the phases requires prolonged waiting time) and the aqueous phase was cooled to 0 °C. Addition of potassium iodide (2.74 g, 16.5 mmol) led to the precipitation of the product, which was filtered off, washed with cold H_2O and dried in a vacuum desiccator to obtain the pure product as an off-white solid (2.66 g, 7.35 mmol, 49% yield). As the desired free base **D** decomposed upon prolonged storage, the compound had to be generated fresh from the ammonium iodide salt prior to use. The salt was suspended in CH_2Cl_2 and equivalent volume of 10% aqueous K_2CO_3 solution was added. The mixture was shaken until the solid had dissolved, after which the organic phase was removed, the aqueous phase extracted twice more with CH_2Cl_2 and the combined organic phases were concentrated *in vacuo* to obtain the free base in quantitative yield. **^{1}H NMR** (500 MHz, $CDCl_3/CD_3OD$ 9:1) δ_H = 7.27-7.22 (m, 2H), 7.12-6.97 (m, 3H), 3.25 (m, 1H), 3.18 (m, 1H), 2.07 (m, 1H), 1.94 (m, 1H), 1.70 (m, 2H), 1.42-1.14 (m, 4H); **^{13}C NMR** (125 MHz, $CDCl_3/CD_3OD$), δ_c = 155.5, 150.5, 128.9, 125.3, 121.4, 54.2, 52.7, 31.3, 29.5, 24.0, 23.4.

2-(dimethylamino)benzaldehyde (2)[4]

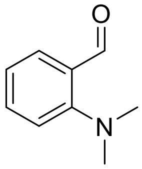

To a suspension of 2-fluorobenzaldehyde (0.55 mL, 646 mg, 5.0 mmol) and K_2CO_3 (691 mg, 5.0 mmol) in anhydrous DMF (10 mL), dimethylamine (5.6 M solution in EtOH, 1.2 mL, 6.7 mmol) was slowly added under N_2 atmosphere at room temperature. The reaction mixture was subsequently stirred at 110 °C for 20 h, at which point the starting material was consumed according to TLC. The bright yellow solution was diluted with ethyl acetate (12 mL) and distilled. H_2O (20 mL) was added, and upon separation of the phases, the aqueous layer was extracted with ethyl acetate (2 x 20 mL) and the combined organic phases were washed with H_2O (40 mL) and brine (40 mL). Drying with $MgSO_4$, filtration and concentration yielded the crude product as a yellow oil. Purification by column chromatography (hexane/ethyl acetate 9:1 → 7:1) yielded the product as a yellow liquid (684 mg, 92% yield). **^{1}H NMR** (500 MHz, $CDCl_3$) δ_H = 10.22 (s, 1H), 7.76 (dd, J = 7.7, 1.6 Hz, 1H), 7.46 (dt, J = 7.8, 1.7 Hz, 1H), 7.05 (d, J = 8.3 Hz, 1H), 7.01 (t, J = 7.5, 1H), 2.92 (s, 3H); **^{13}C NMR** (125 MHz, $CDCl_3$), δ_c = 191.2, 155.7, 134.6, 131.0, 127.0, 120.7, 117.6, 45.5.

2-(diphenylphosphanyl)benzaldehyde (3)[5]

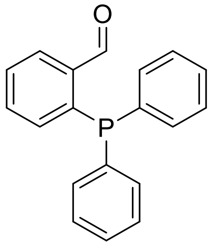

To a vigorously stirred solution of 2-bromobenzaldehyde (2.70 g, 14.6 mmol) and $Pd(PPh_3)_4$ (0.169 g, 0.146 mmol) in anhydrous toluene (50 mL) were sequentially added NEt_3 (2.0 mL, 1.47 g, 14.6 mmol) and diphenylphosphine (3.21 mL, 3.44 g, 19.0 mmol) under N_2 at room temperature. The bright red solution was stirred at reflux for 2.5 h, upon which time the starting material was consumed according to TLC analysis. The milky suspension was filtered through a glass filter, followed by washing with saturated aq. NH_4Cl solution (3 x 40 mL) and brine (40 mL). The solvent was removed in vacuo, yielding the crude product as a yellow solid that was recrystallized twice from MeOH to obtain the pure product as yellow crystals (2.63 g, 62% yield). **^{1}H NMR** (500 MHz, $CDCl_3$) δ_H = 10.50 (d, *J* = 5.4 Hz, 1H), 8.00-7.96 (m, 1H), 7.52-7.45 (m, 2H), 7.38-7.26 (m, 10H), 6.98-6.96 (m, 1H); **^{13}C NMR** (125 MHz, $CDCl_3$), δ_c = 191.7 (d, *J* = 32 Hz), 141.1 (d, *J* = 26 Hz), 138.4 (d, *J* = 14 Hz), 136.1 (d, *J* = 10 Hz), 134.0 (d, *J* = 20 Hz), 133.9, 133.6, 130.6 (d, *J* = 4 Hz), 129.1, 128.8. 128.7 (d, *J* = 7 Hz); **^{31}P NMR** (202 MHz, $CDCl_3$) δ_P = -11.6.

1-methyl-1*H*-imidazole-2-carbaldehyde (4)[6]

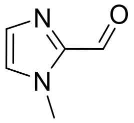

To a solution of *N*-methyl imidazole (1.50 mL, 1.55 g, 18.8 mmol) in anhydrous THF (30 mL) at -78 °C, *n*-butyl lithium (1.6 M solution in hexanes, 17.7 mL, 28.3 mmol) was added dropwise via cannula over 20 minutes under N_2. After stirring for 75 minutes, anhydrous DMF (4.4 mL, 4.13 g, 56.5 mmol) was added dropwise over 20 minutes via syringe and the mixture was further stirred for 4 h at -78 °C. After this time, the milky solution was slowly warmed to room temperature and stirred for an additional 15 h. The reaction was quenched by slow addition of distilled H_2O (30 mL) under heavy stirring and the aqueous layer was extracted with CH_2Cl_2 (3 x 150 mL). The combined organic phases were dried with Na_2SO_4, filtered and concentrated to obtain a yellow oil. Further purification by column chromatography (hexane/ethyl acetate 1:2) yielded the product (1.94 g, 17.6 mmol, 94% yield) as a slightly yellow oil that solidified upon storage. **^{1}H NMR** (500 MHz, $CDCl_3$) δ_H = 9.80 (s, 1H), 7.25 (s, 1H), 7.09 (s, 1H), 4.00 (s, 3H); **^{13}C NMR** (125 MHz, $CDCl_3$), δ_c = 182.2, 143.7, 131.5, 127.3, 34.9.

(*E*)-*N*-benzylidene-4-methylbenzenesulfonamide (6)[7]

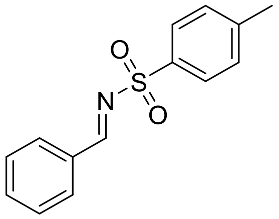

To a mixture of p-toluenesulfonylamide (1.20 g, 7.0 mmol) and pre-activated 4 Å molecular sieves (ca 1 g) in anhydrous toluene (10 mL) were sequentially added benzaldehyde (0.71 mL, 0.743 g, 7.0 mmol) and Amberlyst-15 (0.100 g). The heterogeneous reaction mixture was stirred at reflux under N_2 for 19 h, at which point the starting material was consumed. The reaction was worked up by filtering through a pad of Celite™, followed by repeated washings of the residue with anhydrous toluene and concentration *in vacuo* yielded the almost pure product as a white solid. Further purification was performed by trituration with diethyl ether, yielding the product as a white solid

(1.29 g, 5.0 mmol, 71% yield). ^{1}**H NMR** (500 MHz, $CDCl_3$) δ_H = 9.03 (s, 1H), 7.93 (d, J = 7.3 Hz, 2H), 7.89 (d, J = 8.2 Hz, 2H), 7.62 (t, J = 7.4 Hz, 1H), 7.49 (t, J = 7.7 Hz, 2H), 7.35 (d, J = 8.2 Hz, 2H), 2.44 (s, 3H); ^{13}**C NMR** (125 MHz, $CDCl_3$), δ_c = 170.1, 144.6, 135.1, 134.9, 132.4, 131.3, 129.8, 129.1, 128.1, 21.6.

SYNTHESIS AND CHARACTERIZATION OF IMINE CATALYSTS

General synthetic procedure for imine condensation reactions

To a mixture of amine (0.4 mmol) and pre-activated 4 Å molecular sieves (ca 0.8 g) in anhydrous CH_2Cl_2 (10 mL) was added the aldehyde (0.4 mmol) under N_2. The reaction mixture was slowly stirred overnight, and monitored by sampling of a small aliquot of the reaction mixture which was analyzed by NMR spectroscopy. Upon completion of reaction, the mixture was filtered through a pad of Celite and concentrated *in vacuo* to obtain the imine product with a typical purity >98%.

(*E*)-*N*-cyclohexyl-1-phenylmethanimine (A1)[9]

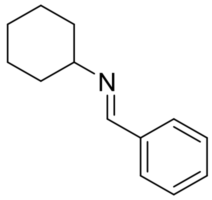

Colorless oil, 87% yield. ^{1}**H-NMR** (500 MHz, $CDCl_3$) δ_H = 8.32 (s, 1H), 7.74-7.71 (m, 2H), 7.41-7.38 (m, 3H), 3.20 (dt, J = 10.6, 4.1 Hz, 1H), 1.87-1.81 (m, 2H), 1.76-1.66 (m, 3H), 1.60 (dq, J = 12.8, 2.5 Hz, 2H), 1.42-1.32 (m, 2H), 1.31-1.22 (m, 1H); ^{13}**C-NMR** (125 MHz, $CDCl_3$) δ_C = 158.6, 136.6, 130.3, 128.5, 128.0, 70.0, 34.5, 25.6, 24.8.

(*E*)-2-((cyclohexylimino)methyl)-*N,N*-dimethylaniline (A2)[10]

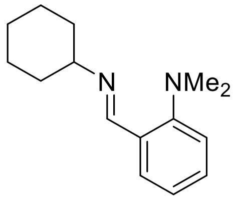

Yellow oil, 88% yield. ^{1}**H-NMR** (500 MHz, $CDCl_3$) δ_H = 8.62 (s, 1H), 7.84 (dd, J = 7.6, 1.2 Hz, 1H), 7.32 (td, J = 7.6, 1.5 Hz, 1H), 7.04-6.99 (m, 2H), 3.26-3.19 (m, 1H), 2.76 (s, 6H), 1.87-1.81 (m, 2H), 1.78-1.73 (m, 2H), 1.70-1.65 (m, 1H), 1.60 (qd, J = 12.2, 2.6 Hz, 2H), 1.42-1.32 (qt, J = 12.7, 3.1 Hz, 2H), 1.30-1.21 (m, 1H); ^{13}**C-NMR** (125 MHz, $CDCl_3$) δ_C =157.8, 153.5, 130.6, 129.5, 128.4, 122.3, 117.7, 70.0, 45.3, 34.5, 25.7, 24.9.

***(E)-N*-cyclohexyl-1-(2-(diphenylphosphanyl)phenyl)methanimine (A3)**

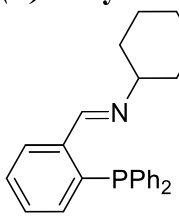

The compound was synthesized according to the general procedure, but with 1.1 equivalents of cyclohexylamine which was removed after reaction by prolonged concentration in vacuo under mild heating. Colorless oil, solidifies to white solid upon storage, 94% yield. ^{1}**H-NMR** (500 MHz, $CDCl_3$) δ_H = 8.81 (d, J = 4.7 Hz, 1H), 7.89 (dd, J = 7.1, 3.8 Hz, 1H), 7.32-7.16 (m, 12H), 6.77 (dd, J = 7.0, 5.2 Hz, 1H), 2.98 (td, J = 10.2, 5.1 Hz, 1H), 1.67-1.61 (m, 2H), 1.55-1.49 (m, 1H), 1.44-1.38 (m, 2H), 1.32 (td, J = 12.7, 3.0 Hz, 2H), 1.23-1.07 (m, 3H); ^{13}**C-NMR** (125 MHz, $CDCl_3$) δ_C = 157.2 (d, J = 21.4 Hz), 139.9 (d, J = 17.1 Hz), 137.0 (d, J = 18.7 Hz), 136.5 (d, J = 9.5 Hz), 134.0

(d, J = 20.0 Hz), 133.1, 129.9, 128.9, 128.7, 128.52 (d, J = 7.2 Hz), 69.5, 34.1, 25.6, 24.7; **^{31}P-NMR** (202 MHz, $CDCl_3$) δ_P = -13.8; **HRMS** found 372.1873, calc. for $C_{25}H_{26}NP$ [M+H$^+$] 372.1876.

1-((±)-2-(((*E*)-benzylidene)amino)cyclohexyl)-3-(3,5-bis(trifluoromethyl)phenyl)thiourea (B1)[11]

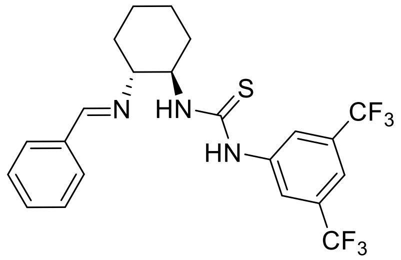

83% yield, white solid. **^{1}H-NMR** (500 MHz, $CDCl_3$) δ_H = 9.71 (s, br, 1H), 8.38 (s, 1H), 7.74 (s, 2H), 7.70-7.66 (m, 2H), 7.57 (s, 1H), 7.51-7.48 (m, 1H), 7.44-7.39 (m, 2H), 6.25 (d, br, *J* = 8.8 Hz, 1H), 3.92-3.85 (m, 1H), 3.18 (td, *J* = 6.7, 12.7 Hz, 1H), 2.23-2.18 (m, 1H), 1.93-1.81 (m, 2H), 1.74-1.63 (m, 2H), 1.50-1.37 (m, 3H); **^{13}C-NMR** (125 MHz, $CDCl_3$) δ_C = 183.2, 163.8, 141.2, 134.8, 132.1, 131.5 (q, *J* = 33 Hz), 129.1, 128.2, 124.7 (br), 123.1 (q, *J* = 271 Hz), 118.4 (br), 75.7, 60.2, 34.0, 31.9, 24.7, 23.9.

1-(3,5-bis(trifluoromethyl)phenyl)-3-((±)-2-(((*E*)-2-(dimethylamino)benzylidene)amino)cyclohexyl)thiourea (B2)

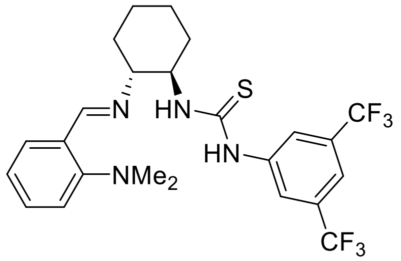

Off-white solid, 90% yield. **^{1}H-NMR** (500 MHz, $CDCl_3$) δ_H = 10.2 (s, br, 1H), 8.66 (s, 1H), 7.75 (d, *J* = 6.7 Hz, 1H), 7.71 (s, 2H), 7.50 (s, 1H), 7.42 (t, *J* = 7.2 Hz, 1H), 7.08-7.02 (m, 2H), 6.33 (d, br, *J* = 7.0 Hz, 1H), 3.88-3.81 (m, 1H), 3.20 (td, *J* = 10.7, 4.6 Hz, 1H), 2.74 (s, 6H), 2.19-2.16 (m, 1H), 1.93-1.82 (m, 2H), 1.78-1.63 (m, 2H), 1.48-1.34 (m, 3H); **^{13}C-NMR** (125 MHz, $CDCl_3$) δ_C = 183.0, 162.9, 154.5, 141.4, 132.7, 131.4 (q, *J* = 33 Hz), 128.0, 127.5, 123.9 (br), 123.1 (q, *J* = 271 Hz), 122.3, 118.4, 117.8 (br), 74.2, 60.2, 45.4, 33.7, 31.7, 24.8, 24.0; **^{19}F-NMR** (376 MHz, without proton decoupling, $CDCl_3$) δ_F = -62.9 (s); **HRMS** found 517.1842, calc. for $C_{24}H_{26}F_6N_4S$ [M+H$^+$] 517.1855.

1-(3,5-bis(trifluoromethyl)phenyl)-3-((±)-2-((*E*)-2-(diphenylphosphanyl)benzylidene)amino)cyclohexyl)thiourea (B3)

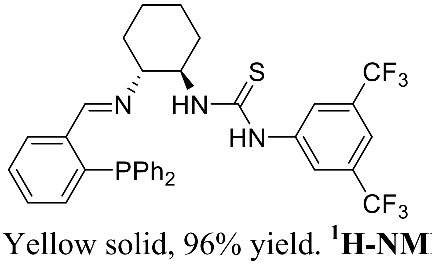

Yellow solid, 96% yield. **^{1}H-NMR** (500 MHz, $CDCl_3$) δ_H = 9.96 (s, br, 1H), 8.86 (d, *J* = 3.9 Hz, 1H), 7.81-7.77 (m, 1H), 7.69 (s, 2H), 7.59 (s, 1H), 7.37-7.28 (m, 8H), 7.22 (t, *J* = 7.1 Hz, 2H), 7.17 (t, *J* = 7.4 Hz, 2H), 6.91-6.87 (m, 1H), 6.16 (d, br, *J* = 7.0 Hz, 1H), 3.79-3.70 (m, br, 1H), 3.02-2.95 (m, 1H), 2.15-2.09 (m, 1H), 1.85-1.80 (m, 1H), 1.73-1.68 (m, 1H), 1.44-1.24 (m, 5H); **^{13}C-NMR** (125 MHz, $CDCl_3$) δ_C = 183.1, 162.7 (d, *J* = 18.6 Hz), 141.5, 138.2 (dd, *J* = 18.4, 14.9 Hz), 135.7 (dd, *J* = 8.4, 5.2 Hz), 134.3 (d, *J* = 20.3 Hz), 133.8, 133.5 (d, *J* = 19.7 Hz), 131.5 (q, *J* = 23.2 Hz), 131.4, 129.2 (d, *J* = 4.0 Hz), 129.0, 128.7 (dd, *J* = 14.3, 7.4 Hz), 127.9 (d, *J* = 4.0 Hz), 124.9, 123.1

(q, J = 271.3 Hz), 118.3 (br), 71.5, 60.2, 33.5, 31.9, 24.6, 23.9; **^{19}F-NMR** (376 MHz, without proton decoupling, $CDCl_3$) δ_F = -62.7; **^{31}P-NMR** (202 MHz, $CDCl_3$) δ_P = -12.5; **HRMS** found 658.1864, calc. for $C_{34}H_{30}F_6N_3PS$ [M+H$^+$] 658.1875.

1-((±)-2-(((*E*)-2-(diphenylphosphanyl)benzylidene)amino)cyclohexyl)-3-phenylthiourea (C3)

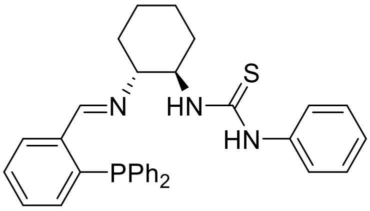

White solid, 96% yield. This compound experiences severe peak broadening in NMR measurements, possibly due to aggregation effects. **^{1}H-NMR** (500 MHz, $CDCl_3$) δ_H = 9.34 (s, br, 1H), 8.73 (d, J = 3.8 Hz, 1H), 8.00-7.75 (m, br, 1H), 7.35-6.96 (m, 17H), 6.81-6.76 (m, br, 1H), 5.94-5.70 (s, br, 1H), 4.40-3.56 (m, br, 1H), 2.88 (dd, J = 14.9, 9.5 Hz, 1H), 2.34-2.05 (m, br, 1H), 1.74-1.53 (m, 2H), 1.35-1.10 (m, 5H); **^{13}C-NMR** (125 MHz, $CDCl_3$) δ_C = 182.3 (br), 160.8 (br), 137.7 (br), 137.2, 137.1, 136.2 (d, J = 10.3 Hz), 136.2 (d, J = 7.9 Hz), 134.1 (d, br J = 17.4 Hz), 133.7, 133.6, 133.5, 130.7, 128.9 (br), 128.6 (dd, J = 13.2, 7.2 Hz), 128.4 (d, J = 6.8 Hz), 125.7, 74.5 (br), 59.5, 33.2, 31.6, (br), 24.5, 23.0; **^{31}P-NMR** (202 MHz, $CDCl_3$) δ_P = [-11.0]- [-15.1] (m, br); **HRMS** found 522.2122, calc. for $C_{32}H_{32}N_3PS$ [M+H$^+$] 522.2127.

Phenyl ((±)-2-(((*E*)-2-(diphenylphosphanyl)benzylidene)amino)cyclohexyl)carbamate (D3)

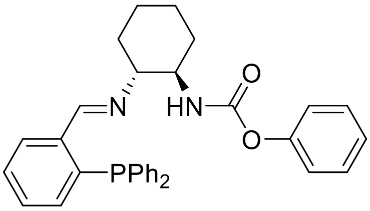

The compound was synthesized from the hydroiodide salt of compound **D** by a modified general procedure. To a suspension of **D•HI** (74.6 mg, 0.206 mmol) and 4 Å molecular sieves (ca 400 mg) in anhydrous CH_2Cl_2 (5 mL) was added the aldehyde **3** (58.1 mg, 0.20 mmol) followed by addition of freshly distilled NEt_3 (27.9 μL, 20.2 mg, 0.20 mmol). The milky solution immediately turned clear, and the mixture was stirred at rt for 19 h. The solution was then filtered through a pad of celite and washed with H_2O (2x10 mL) and brine (10 mL). Drying with $MgSO_4$, filtration and concentration yielded a white mixture which was recrystallized from MeOH to obtain the product as white crystals (52.7 mg, 52%). **^{1}H-NMR** (500 MHz, $CDCl_3$) δ_H = 8.88 (d, J = 4.7 Hz, 1H), 8.01 (dd, J = 7.1, 3.7 Hz, 1H), 7.40 (t, J = 7.5 Hz, 1H), 7.35-7.23 (m, 13H), 7.13 (t, J = 7.1 Hz, 1H), 6.93 (s, br, 1H), 6.87 (dd, J = 7.0, 5.2 Hz, 1H), 4.62-4.55 (m, br, 1H), 3.65-3.58 (m, br, 1H), 3.00-2.91 (m, br, 1H), 2.17 (d, J = 11.1 Hz, 1H), 1.73 (t, J = 15.1 Hz, 2H), 1.58-1.47 (m, 2H), 1.40-1.23 (m, 3H); **^{13}C NMR** (125 MHz, $CDCl_3$), δ_c = 159.2 (d, J = 20.0 Hz), 151.0, 139.3 (d, J = 15.7 Hz), 137.3 (d, J = 19.1 Hz), 136.6 (t, J = 9.0 Hz), 134.0 (d, J = 19.9 Hz), 133.3, 130.3, 129.1, 129.0, 128.8 (d, J = 7.7 Hz), 128.61 (d, J = 7.0 Hz, overlap), 128.58 (d, J = 7.1 Hz, overlap), 128.2 (br), 125.0, 121.6, 73.9, 55.2, 33.2, 31.4, 24.8, 24.1; **^{31}P-NMR** (202 MHz, $CDCl_3$) δ_P = -13.4; **HRMS** found 507.2185, calc. for $C_{32}H_{31}N_2O_2P$ [M+H$^+$] 507.2196.

Experimental procedure for Morita-Baylis-Hillman reactions

2-(hydroxy(4-nitrophenyl)methyl)pent-1-en-3-one (5)[12]

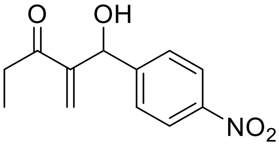

To a solution of *p*-nitrobenzaldehyde (30.2 mg, 0.2 mmol) and compound **C3** (20.9 mg, 0.04 mmol) in anhydrous THF (1.0 mL) was added ethyl vinyl ketone (59.7 μL, 50.5 mg, 0.6 mmol) and 4 Å

MS (100 mg), and the yellow solution was stirred at room temperature. After 240 h, the reaction had stopped according to NMR analysis. Yield 87%, based on NMR analysis with 1,4-dimethoxybenzene as internal standard. The compound was isolated by filtering through a glass filter, then concentrating the solution under reduced pressure followed by purification by column chromatography (hexane/ethyl acetate 3:1) to obtain the product as a colorless oil (33.9 mg, 72%). **^{1}H-NMR** (500 MHz, $CDCl_3$) δ_H = 8.19 (d, *J* = 8.7 Hz, 2H), 7.55 (d, *J* = 8.7 Hz, 2H), 6.26 (s, 1H), 5.99 (s, 1H), 5.67 (d, *J* = 4.8 Hz, 1H), 3.37 (d, *J* = 5.6 Hz, 1H), 2.73 (q, *J* = 7.3 Hz, 2H), 1.06 (t, *J* = 7.3 Hz, 3H); **^{13}C-NMR** (125 MHz, $CDCl_3$) δ_C = 202.9, 149.0, 148.4, 147.3, 127.2, 126.4, 123.6, 72.7, 31.4, 7.9.

4-methyl-*N*-(2-methylene-3-oxo-1-phenylpentyl)benzenesulfonamide (7)[13]

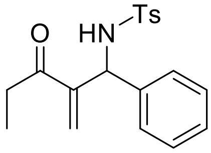

To a solution of (*E*)-*N*-benzylidene-4-methylbenzenesulfonamide **(6)** (51.9 mg, 0.2 mmol) and compound **C3** (20.9 mg, 0.04 mmol) in anhydrous THF (1.0 mL) was added ethyl vinyl ketone (59.7 µL, 50.5 mg, 0.6 mmol) and 4 Å MS (100 mg), and the yellow solution was stirred at room temperature. After 72 h, the reaction was finished according to NMR analysis. Yield 85%, based on NMR measurement with 1,4-dimethoxybenzene as internal standard. The compound was isolated by filtering through a pad of celite, then concentrating the solution under reduced pressure followed by purification by column chromatography (hexane/ethyl acetate 4:1) to obtain the product as a yellow oil that solidified upon storage (52.8 mg, 77%). **^{1}H-NMR** (500 MHz, $CDCl_3$) δ_H = 7.65 (d, *J* = 8.1 Hz, 1H), 7.24-7.18 (m, 5H), 7.10 (d, *J* = 7.4 Hz, 1H), 6.09 (s, 1H), 6.04 (s, 1H), 5.68 (s, br, 1H), 5.27 (s, 1H), 2.60-2.52 (m, 1H), 2.47-2.42 (m, 1H), 2.40 (s, 3H), 0.92 (t, *J* = 7.2 Hz, 2H); **^{13}C-NMR** (125 MHz, $CDCl_3$) δ_C = 201.5, 145.9, 143.3, 138.9, 137.6, 129.4, 128.5, 127.6, 127.3, 126.9, 126.3, 59.3, 31.3, 21.5, 7.8.

INVESTIGATION OF TRANSIMINATION CATALYSIS BY THIOUREA

Model system generation

Cyclohexylamine (11.4 µL, 9.9 mg, 0.10 mmol), thiourea **B** (38.5 mg, 0.10 mmol), 2-dimethylaminobenzaldehyde (14.9 mg, 0.10 mmol) and benzaldehyde (10.1 µL, 10.6 mg, 0.10 mmol) were dissolved in anhydrous THF (2.5 mL) with pre-activated 4 Å MS (300 mg), and the mixture was stirred slowly at room temperature for 20 h. The system generation was monitored by sampling of an aliquot (20 µL) of the reaction mixture, which was dissolved in anhydrous $CDCl_3$ (0.55 mL) and analyzed by NMR spectroscopy. After the full system was generated, four new imines with clear separate singlets in the ^{1}H NMR spectrum (δ_H = 8.65, 8.61, 8.37, 8.31 ppm) were obtained (see Figure S1).

Equilibrium manipulation

To probe the dynamic system equilibrium, the drying agent was initially removed by filtration. To the resulting solution, H_2O (0.01 equiv.) and benzoic acid (0.05 equiv.) dissolved in anhydrous THF (60 µL) were added and the system was stirred under N_2 at room temperature. Subsequent composition controls by NMR spectroscopy according to the description above revealed that no significant change in system composition (only minor hydrolysis occurred) had taken place after either 24 or 48 h. Similar results were also reached when $Sc(OTf)_3$ (0.05 equiv.) was used as equilibration catalyst. The following system distribution was recorded after 48 h:

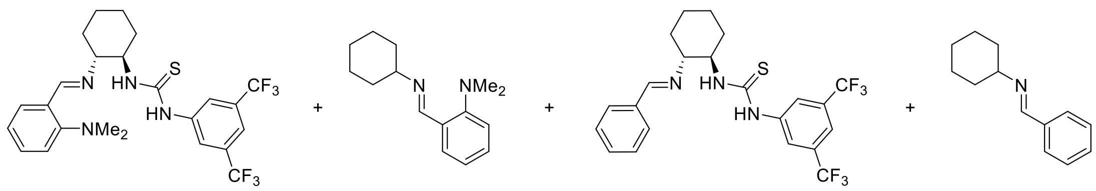

Equilibrium composition: 29% 22% 16% 36%

To investigate whether the system had reached equilibrium, or if the imine exchange had been inhibited, salicylaldehyde (10.5 µL, 12.2 mg, 0.10 mmol) was added to the colorless solution. Instant imine exchange occurred, as evidenced by the appearance of a strongly yellow color corresponding to the newly generated salicylimines. The system was monitored by NMR spectroscopy and reached a new stable equilibrium within 48-72 hours, showing evident dynamic behavior.

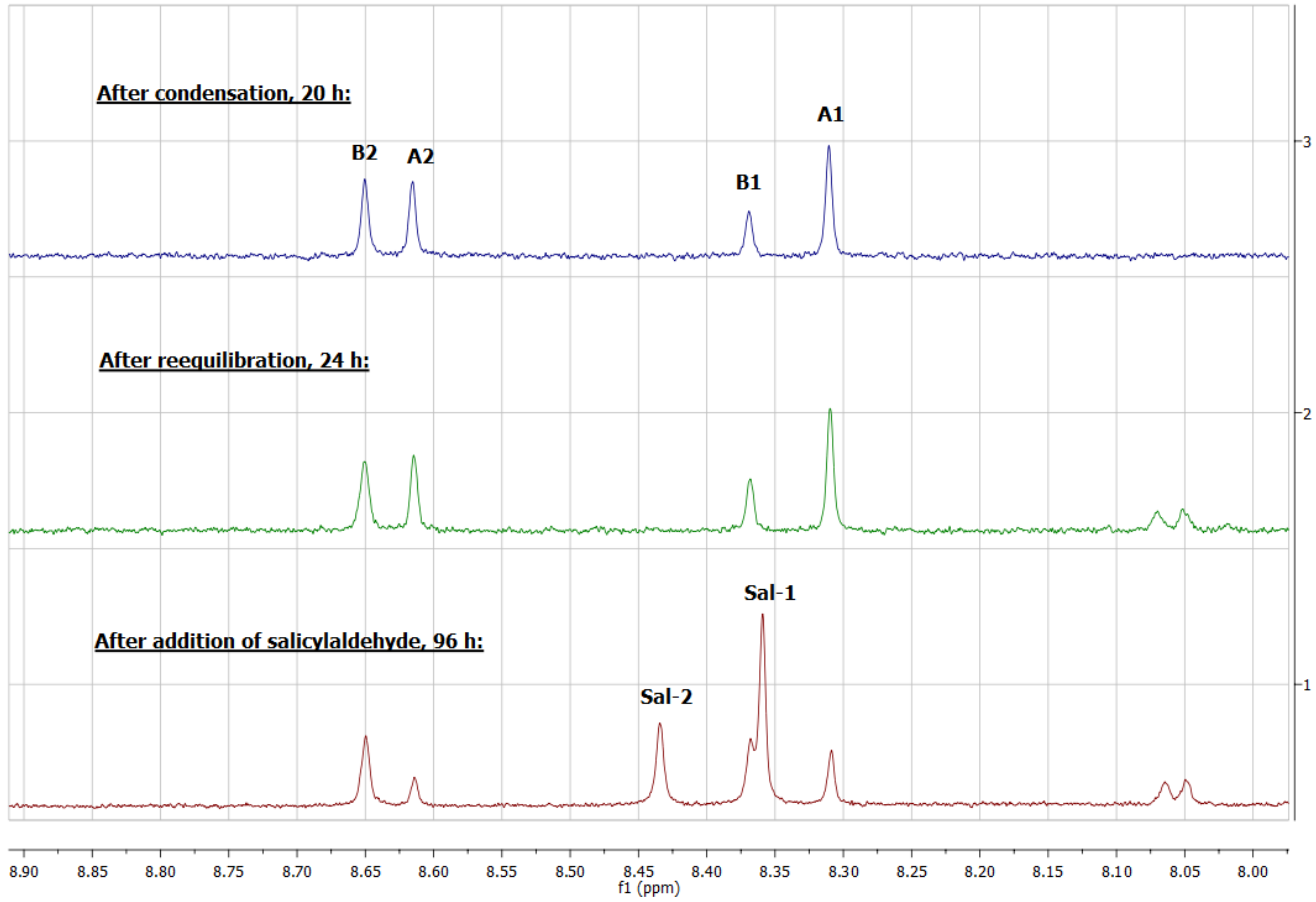

Figure S1. Model system obtained after direct condensation (top), after reequilibration with benzoic acid for 24 h (middle) and 96h after addition of salicylaldehyde to the reaction mixture (bottom).

Thiourea-catalyzed condensation and equilibration

To confirm that the system was capable of catalyzing the equilibration of its own members during the condensation phase, the final equilibrium investigated above was also approached in a single step by mixing the three aldehydes directly with the two amines. Cyclohexylamine (11.4 µL, 9.9 mg, 0.10 mmol), thiourea **B** (38.5 mg, 0.10 mmol), 2-dimethylaminobenzaldehyde (14.9 mg, 0.10 mmol), benzaldehyde (10.1 µL, 10.6 mg, 0.10 mmol) and salicylaldehyde (10.5 µL, 12.2 mg, 0.10 mmol) were dissolved in anhydrous THF (2.5 mL) with pre-activated 4 Å MS (300 mg), and the mixture was stirred slowly at room temperature for 20 h. The system generation was monitored by sampling of an aliquot (20 µL) of the reaction mixture, which was dissolved in anhydrous $CDCl_3$

(0.55 mL) and analyzed by NMR spectroscopy. The system composition was highly similar to that obtained through the two-step condensation/re-equilibration procedure, and no change in the ratio of imines was seen after an additional 48 h of stirring. These results thus implicate that equilibration is fast enough to occur during the imine formation phase at this concentration.

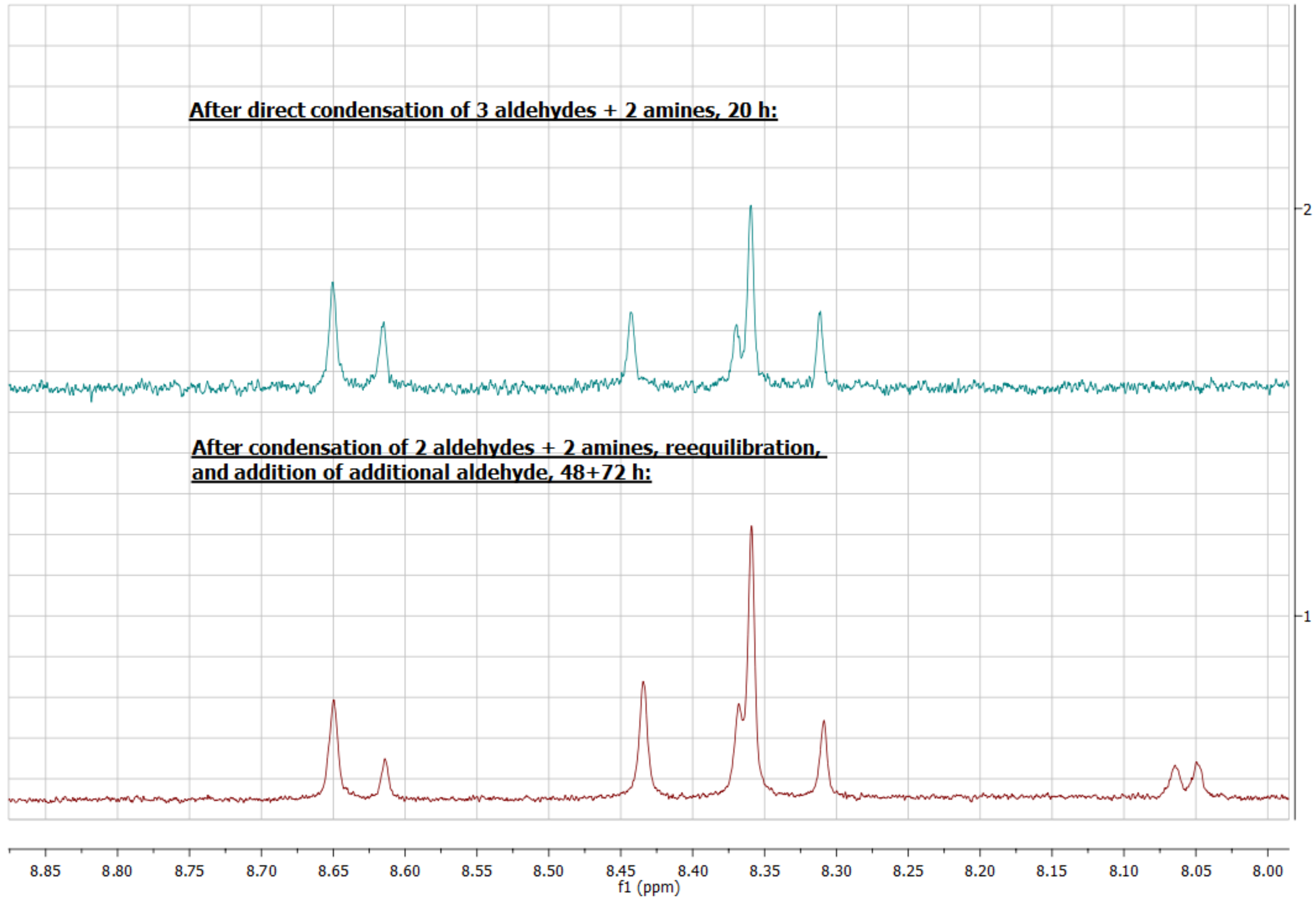

Figure S2. Equilibrium composition from system with benzaldehyde, salicylaldehyde, 2-dimethylaminobenzaldehyde, cyclohexylamine and thiourea-amine **B**, reached in a direct (top) or indirect two-step (bottom) manner.

H_2O-dependence on thiourea-transimination

To investigate if dynamic exchange was still possible in absence of water, the equilibrated system generated in the two-step fashion described above was stirred with 4 Å MS for a further 24 h to ensure complete drying. Afterwards, *p*-nitrobenzaldehyde (15.1 mg, 0.10 mmol) was added to the equilibrated system and NMR measurements were performed after 4 and 24 h. No exchange was observed, suggesting that thiourea-catalyzed transimination is only possible while water is freely available in the system.

Further tests for transimination catalysis by thiourea

To investigate whether only the strongly H-bond-donating, electron-deficient thiourea **B** and its imine derivatives was capable of catalyzing the imine equilibration, or if the catalytic functionality could be extended to other H-bond donors, a series of investigations on the privileged catalyst structures **B3**, **C3** and **D3** found in the dynamic deconvolution process was performed. The imine (0.015 mmol) was mixed with *p*-nitrobenzaldehyde (4.5 mg, 0.03 mmol) in anhydrous $CDCl_3$ (0.55 mL) in an NMR tube, and the colorless mixture was regularly monitored by NMR. The residual NMR tube moisture was in this case enough to initiate transimination for both thiourea-imines **B3** and **C3**, as new characteristic NMR signals in the aldehyde and imine regions could be recorded after 4 h. After 24-72 h, an apparent equilibrium was established, yielding a product ratio of approximately 30:70 in favor of the newly formed imine. In contrast, no transimination was

observed for the urethane **D3** even after 24 h. This suggests that the thiourea scaffold is necessary for transimination to occur. Furthermore, it also concludes that nucleophilic catalysis by primary amines is at least not the sole reason for the self-correcting abilities of the systems observed during condensation.

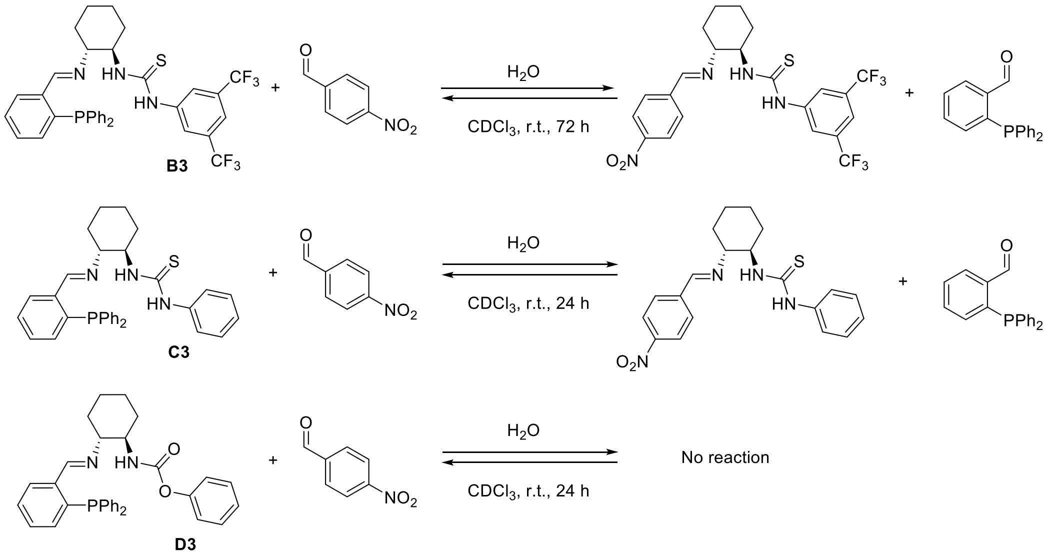

Scheme S1. Thiourea-catalyzed transimination by compounds **B3** and **C3**, and absence of catalytic activity for **D3**.

PROCEDURE FOR SYSTEM GENERATION AND DECONVOLUTION EXPERIMENTS

Experimental procedure for system generation

All aldehydes and amines were dissolved in anhydrous THF (0.5 mL) in an Eppendorf vial and the solution was transferred to a dry vial containing pre-activated 4 Å MS (300 mg, bead diameter ca 2 mm) under N_2. The mixture was stirred slowly at room temperature under N_2 for 20 hours, after which time the equilibrated system was obtained. Tests for thiourea system equilibration were performed (as described in the section above), showing that the systems were at equilibrium after condensation. Also, sensitivity tests were performed, showing that similar system compositions (to within ±5%) were obtained regardless of the order of reagent addition or system member concentration (c=0.04 M, 0.10 M and 0.15 M yielded identical systems, within experimental error). The content of remaining aldehyde was never higher than 5%. The catalyst system could also be isolated by filtering the solution through a pad of celite, concentrating *in vacuo*, and drying at high vacuum for 2 h. The system composition did not change measurably during this procedure.

Experimental procedure for Morita-Baylis-Hillman reactions with dynamic system catalysis

A dynamic system (see Table S1 for amounts, 1 equiv. = 0.075 mmol) was generated according to the description above. After stirring the system building blocks for 20 h under N_2 together with 4 Å MS (ca 300 mg), *p*-nitrobenzaldehyde (18.1 mg, 0.12 mmol) in anhydrous THF (0.120 mL) was added under N_2, followed by addition of ethyl vinyl ketone (23.9 µL, 20.8 mg, 0.24 mmol). The mixture was stirred at room temperature under N_2.

Table S1. Matrix of additions. n = relative amounts

System:	n (**1**)	n (**2**)	n (**3**)	n (**4**)	n (**A**)	n (**B**)	n (**C**)	n (**D**)
Reference	1	1	1	1	1	1	1	1
Replaced **2**	2	0	1	1	1	1	1	1
Replaced **3**	2	1	0	1	1	1	1	1
Replaced **4**	2	1	1	0	1	1	1	1
Replaced **B**	1	1	1	1	2	0	1	1
Replaced **C**	1	1	1	1	2	1	0	1
Replaced **D**	1	1	1	1	2	1	1	0

Comment: Due to the inherent complexity associated with large mixtures of compounds, it was not possible to fully quantify each library component in these reduced libraries. Tests for equilibration were performed and all the mixtures were shown to be at equilibrium. Also, the imine peak region in the ^{1}H NMR spectra of the systems could be used as a spectral fingerprint to determine approximate compound concentrations. This data indicated that no major changes in the relative component distributions seemed to occur upon removal of any of the components in table S1.

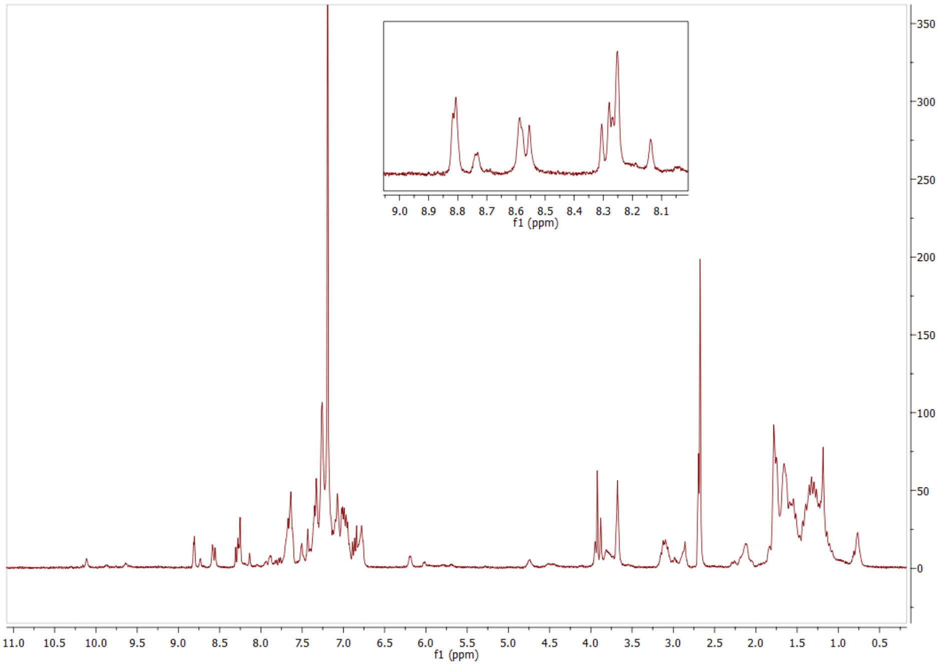

Figure S3. ^{1}H-NMR spectrum of the 16-component reference system, with imine region highlighted.

Kinetic analysis of Morita-Baylis-Hillman reactions

The reaction mixtures were prepared as above and monitored continuously by microsyringe sampling. An aliquot of the reaction mixture (30.0 µL) was added to $CDCl_3$ (0.550 mL) in an NMR tube, with $PhSiMe_3$ (0.020 µL/mL $CDCl_3$) as internal standard. NMR measurements were performed within 5 minutes, though control experiments indicated that the aliquot composition was stable for several hours in anhydrous $CDCl_3$. Product formation was monitored by integrating the two characteristic peaks at around 5.66 and 6.00 and comparing to the integral of the internal standard. As the product concentration in the initial rate measurements were quite low, special precautions for the NMR measurements had to be undertaken. To increase sensitivity, 128 scans for each sample were performed, and the sample was manually processed using TopSpin software by Fourier transforming the FID twice, then performing an automated baseline correction, followed by manual final calibration to make sure the relevant signals were properly aligned. Each integration event was repeated three times and averages are reported.

MBH Catalyst evaluation experiments

To evaluate singular catalyst performance, the 16 different catalysts from the system were synthesized individually and MBH reactions were carried out *in situ*: To a vial under N_2, equipped with 4 Å molecular sieves (200 mg), was added aldehyde (0.02 mmol) and amine (0.02 mmol) by mixing stock solutions (0.1 M) of the respective compound in anhydrous THF. The resulting mixtures were left with slow stirring for 20 h under N_2, after which *p*-nitrobenzaldehyde (15.1 mg, 0.1 mmol) in anhydrous THF (100 µL) and ethyl vinyl ketone (30 µL, 25.3 mg, 0.3 mmol) were added. The solutions were stirred for an additional 24 h, and reaction progress was monitored by removal of an aliquot that was evaluated by NMR spectroscopy with 1,4-dimethoxybenzene as internal standard.

Initial rate data for MBH system catalysis screening

Table S2. Summary of kinetic data for MBH reaction with dynamic systems as catalysts.

Building block replaced	Repetition:	v (a.u.)	v_{rel}	R^2
Ref	1	0,100	1,00	0,963
Ref	2	0,103	1,02	0,962
2	1	0,146	1,45	0,956
2	2	0,100	1,00	0,992
3	1	0,000	0,00	N/A
3	2	0,000	0,00	N/A
4	1	0,100	1,00	0,981
4	2	0,101	1,01	0,995
B	1	0,095	0,94	0,936
B	2	0,180	1,79	0,999
C	1	0,060	0,60	0,988
C	2	0,071	0,71	0,975
D	1	0,131	1,30	0,988
D	2	0,071	0,71	0,999

Experiment # 1:
Replaced building block: None (reference);
Repetition: 1; System: **A**, **B**, **C**, **D**, **1**, **2**, **3**, **4**

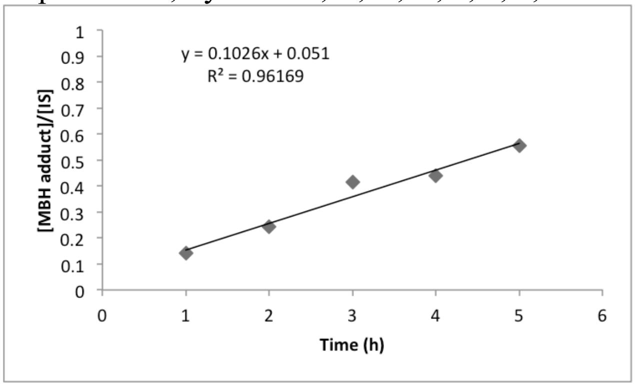

Experiment # 2:
Replaced building block: None (reference);
Repetition: 2; System: **A**, **B**, **C**, **D**, **1**, **2**, **3**, **4**

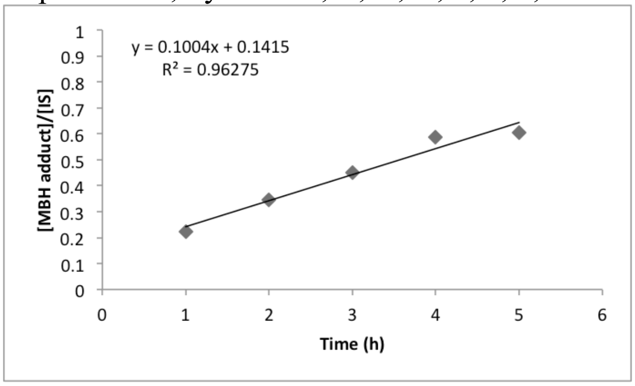

Experiment # 3:
Replaced building block: **2**; Repetition: 1;
System: **A**, **B**, **C**, **D**, **1** (2 equiv.), **3**, **4**

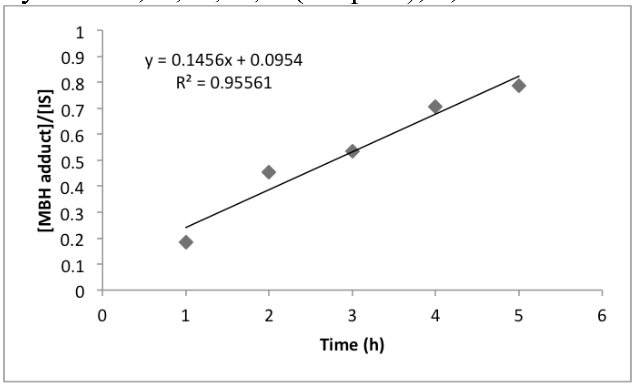

Experiment # 4:
Replaced building block: **2**; Repetition: 2;
System: **A**, **B**, **C**, **D**, **1** (2 equiv.), **3**, **4**

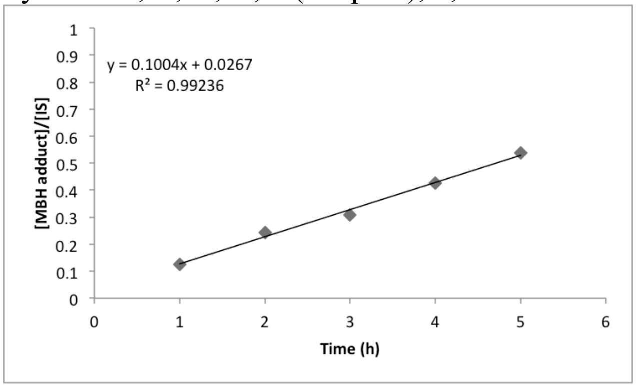

Experiment #5-6:
Replaced building block: **3**
Repetition: 1, 2
System: **A**, **B**, **C**, **D**, **1** (2 equiv.), **2**, **4**

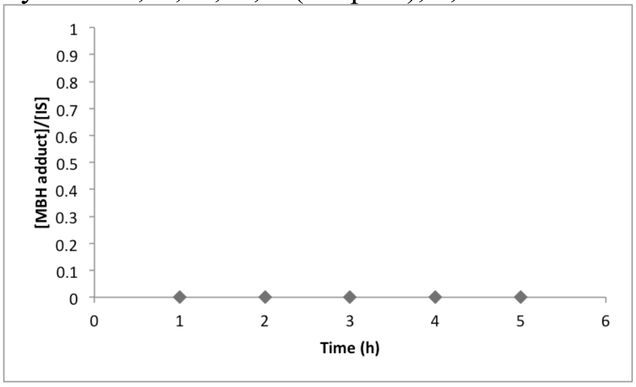

Repeated measurement yielded the same results; removal of compound **3** from the system led to complete suppression of product formation.

Experiment # 7:
Replaced building block: **4**
Repetition: 1
System: **A**, **B**, **C**, **D**, **1** (2 equiv.), **2**, **3**

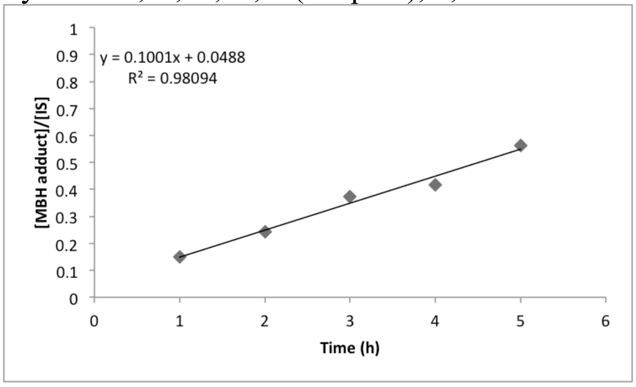

Experiment # 8:
Replaced building block: **4**; Repetition: 2;
System: **A**, **B**, **C**, **D**, **1** (2 equiv.), **2**,**3**

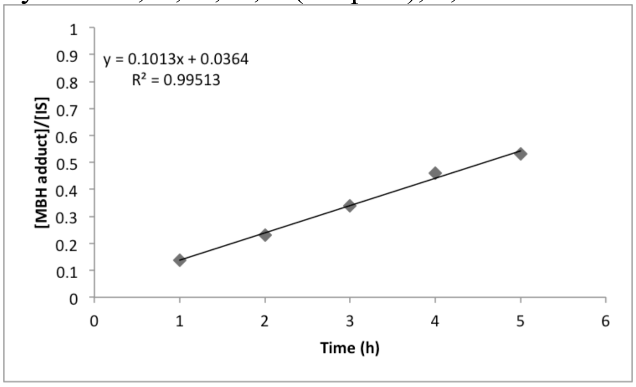

Experiment # 9:
Replaced building block: **B**; Repetition: 1;
System: **A** (2 equiv.), **C**, **D**, **1**, **2**,**3**, **4**

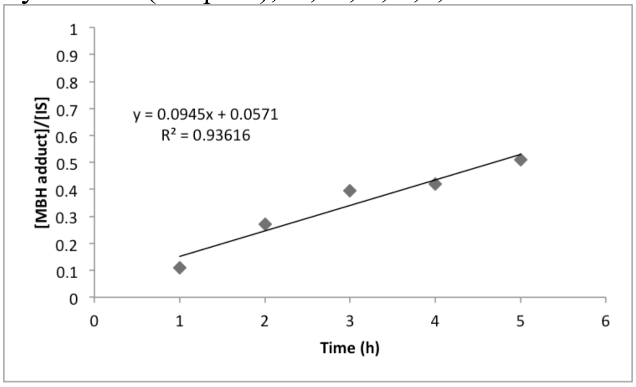

Experiment # 10:
Replaced building block: **B**; Repetition: 2;
System: **A** (2 equiv.), **C**, **D**, **1**, **2**,**3**, **4**

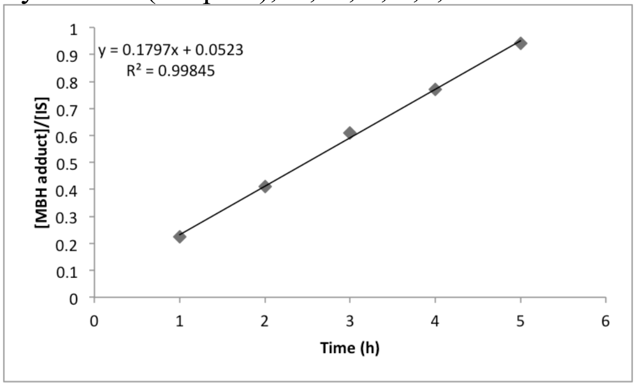

Experiment # 11:
Replaced building block: **C**; Repetition: 1;
System: **A** (2 equiv.), **B**, **D**, **1**, **2**, **3**, **4**

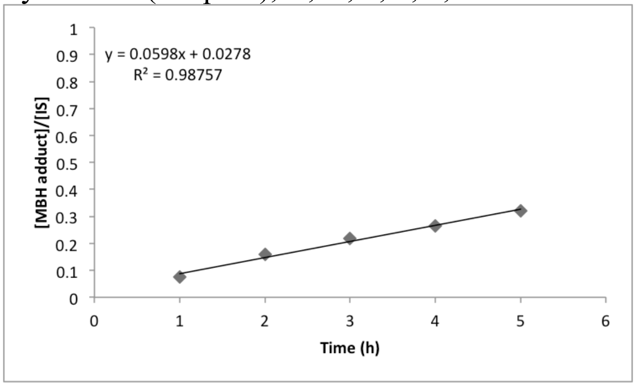

Experiment # 12:
Replaced building block: **C**; Repetition: 2;
System: **A** (2 equiv.), **B**, **D**, **1**, **2**, **3**, **4**

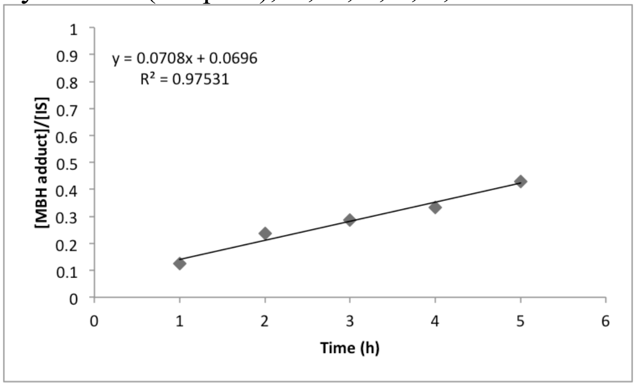

Experiment # 13:
Replaced building block: **D**; Repetition: 1;
System: **A** (2 equiv.), **B**, **C**, **1**, **2**, **3**, **4**

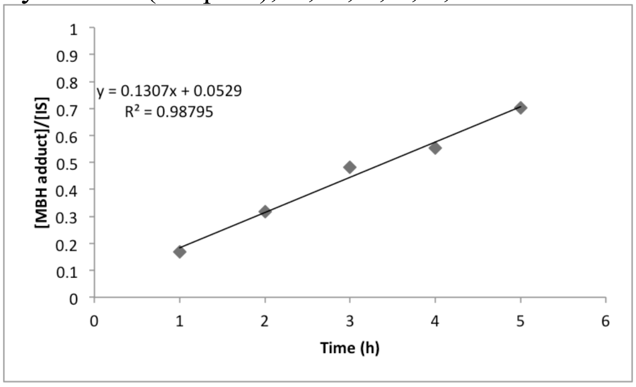

Experiment # 14:
Replaced building block: **D**; Repetition: 2;
System: **A** (2 equiv.), **B**, **C**, **1**, **2**, **3**, **4**

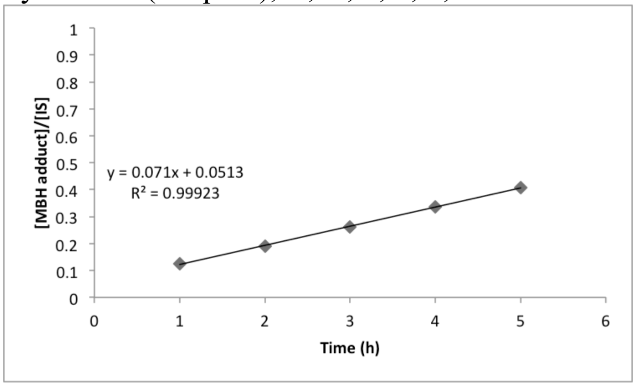

For this system, an initial series revealed almost no reaction. Considering this experiment was not repeatable and not in line with any other data, it was treated as an artefact and omitted.

References

1. X.-j. Zhang, S.-p. Liu, J.-h. Lao, G.-j. Du, M. Yan, A. S. C. Chan, *Tetrahedron Asymmetry*, **2009**, *20*, 12, 1451-1458
2. K. Dudziński, A. M. Pakulska, P. Kwiatkowski, *Org. Lett.*, **2012**, *14*, 4222-4225
3. Á. L. Fuentes de Arriba, D. G. Seisdedos, L. Simón, V. Alcázar, C. Raposo, J. R. Morán, *J. Org. Chem.*, **2010**, *75*, 23, 8303-8306
4. Patent EP2123637, AI, 2009, p. 100
5. S. Laue, L. Greiner, J. Wöltinger, A. Liese, *Adv. Synth. Catal.*, **2001**, *343*, 6-7, 711-720
6. S. E. Howson, L. E. N. Allan, N. P. Chmel, G. J. Clarkson, R. J. Deeth, A. D. Faulkner, D. H. Simpson, P. Scott, *Dalton Trans*., **2011**, *40*, 10416-10433
7. I. Alonso , J. Esquivias , R. Gómez-Arrayás, J. C. Carretero, *J. Org. Chem.*, **2008**, *73*, 16, 6401-6404
8. R. R. Huddleston, H.-Y. Jang, M. J. Krische, *J. Am. Chem. Soc.*, **2002**, *124*, 51, 15156 - 15157
9. H. Tian, X. Yu, Q. Li, J. Wang, Q. Xu, *Adv. Synth. Catal.*, **2012**, *354*, 14-15, 2671–2677
10. G. van Koten, J. G. Noltes , *J. Chem. Soc. Chem. Commun.*, 1972 , 59
11. D. R. Li , A. He, J. R. Falck, *Org. Lett.*, **2010**, *12*, 1756-1759
12. J.-K., Jiang, C.-Q. Li, M. Shi, *Tetrahedron*, 2003 , 59, 8 , 1181-1189
13. L.-H. Chen, C.-Q. Li, M. Shi, *J. Am. Chem. Soc.***, 2005**, 127, 3790-3800

Spectral data for new compounds

Compound A3

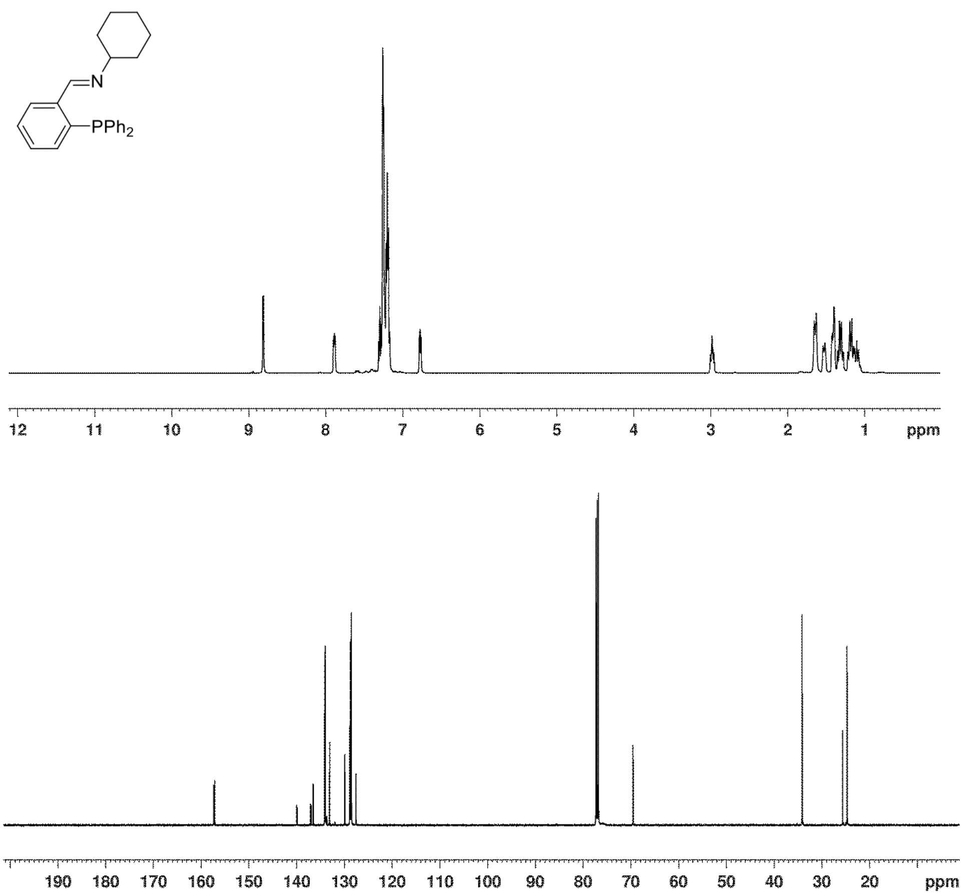

Figure S4. ^{1}H- and ^{13}C-NMR spectra of compound **A3**

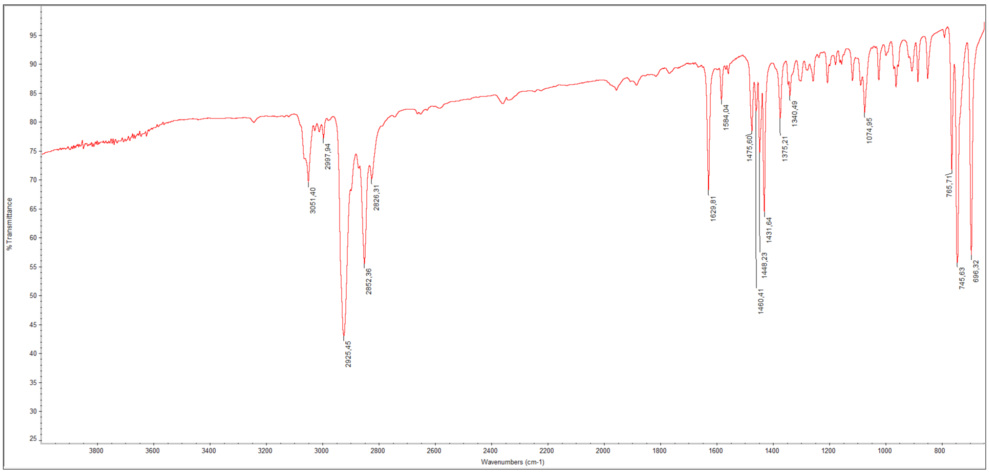

Figure S5. ATR-IR spectrum of compound **A3** (ATR corrected, neat)

Compound B2

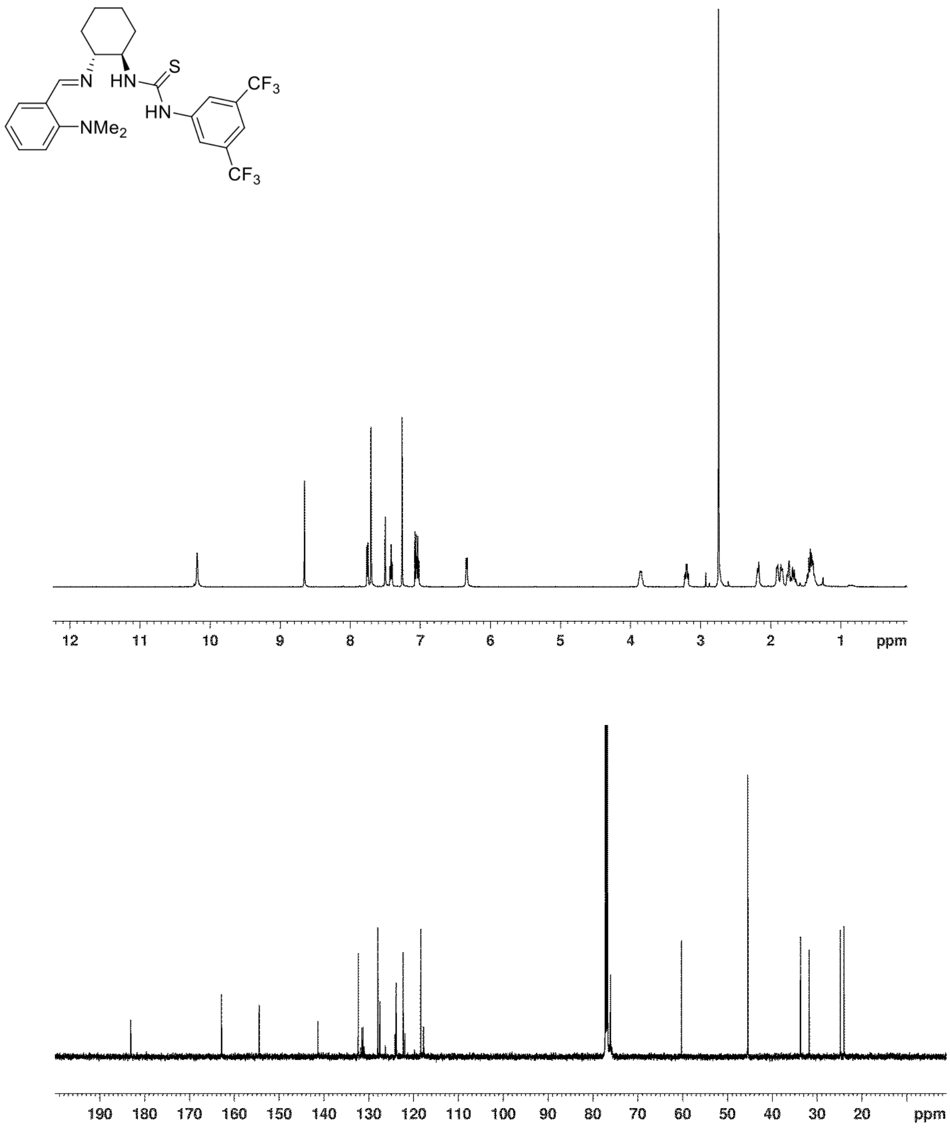

Figure S6. ^{1}H- and ^{13}C-NMR spectra of compound **B2**

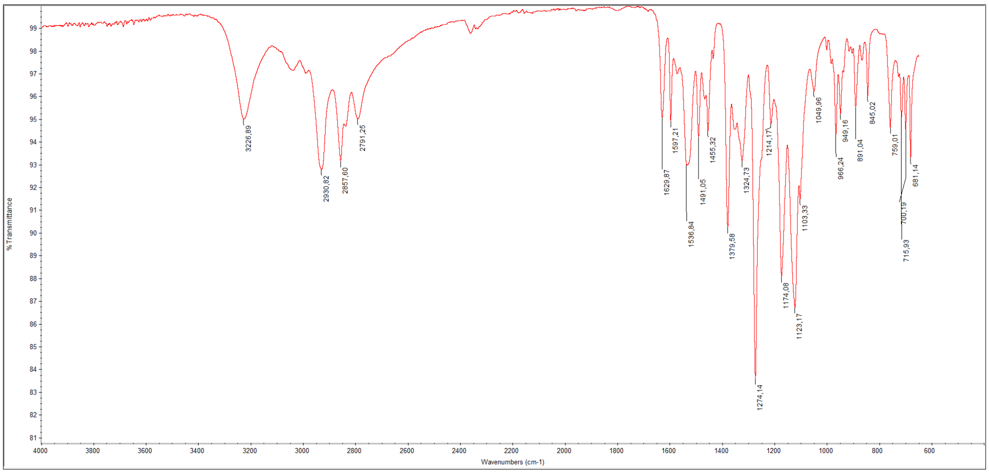

Figure S7. ATR-IR spectrum of compound **B2** (ATR corrected, neat)

Compound B3

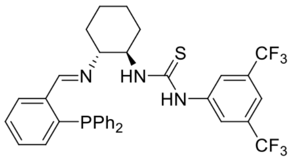

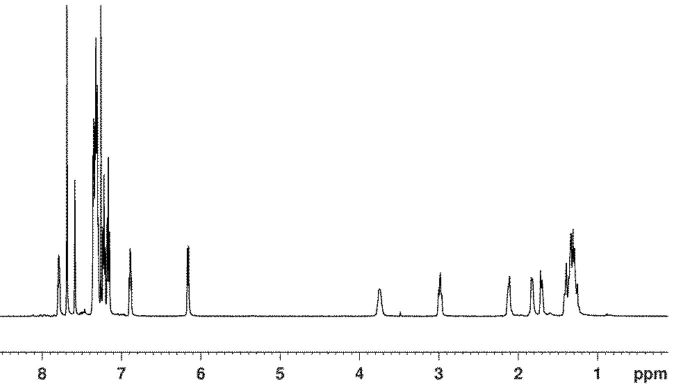

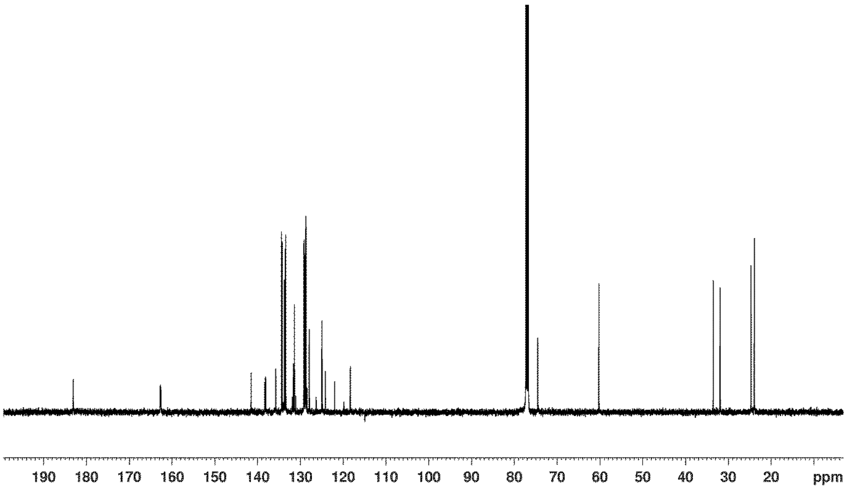

Figure S8. ^{1}H- and ^{13}C-NMR spectra of compound **B3**

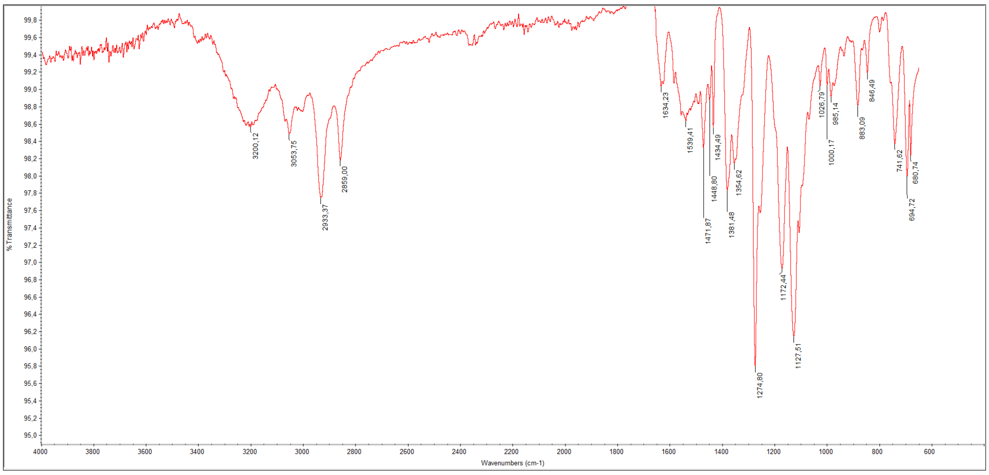

Figure S9. ATR-IR spectrum of compound **B3** (ATR corrected, neat)

Compound C3

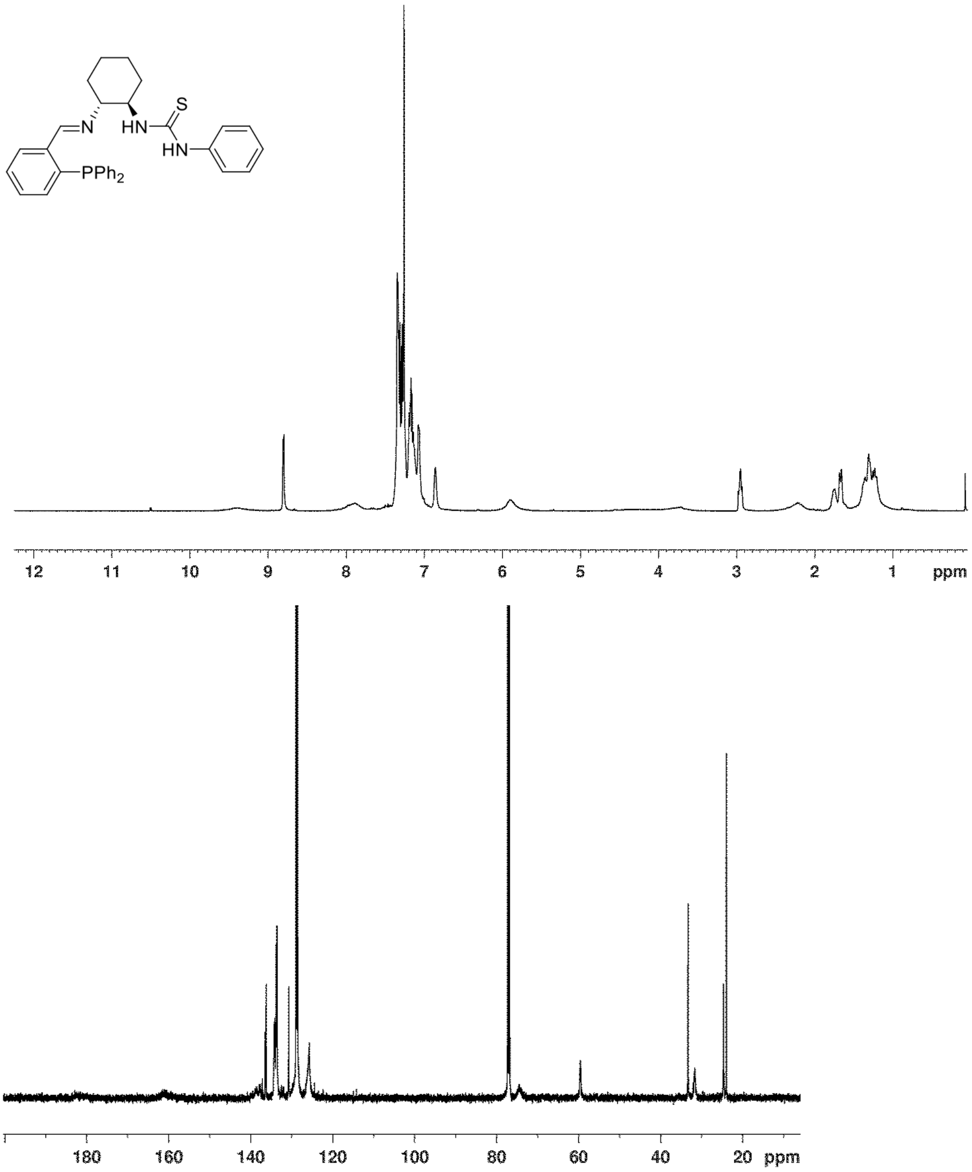

Figure S10. ^{1}H- and ^{13}C-NMR spectra of compound **C3**

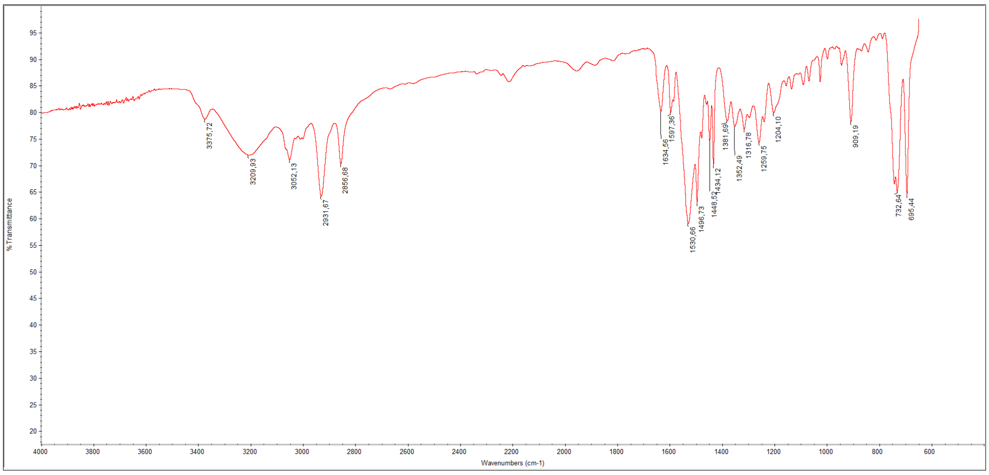

Figure S11. ATR-IR spectrum of compound **C3** (ATR corrected, neat)

Compound D3

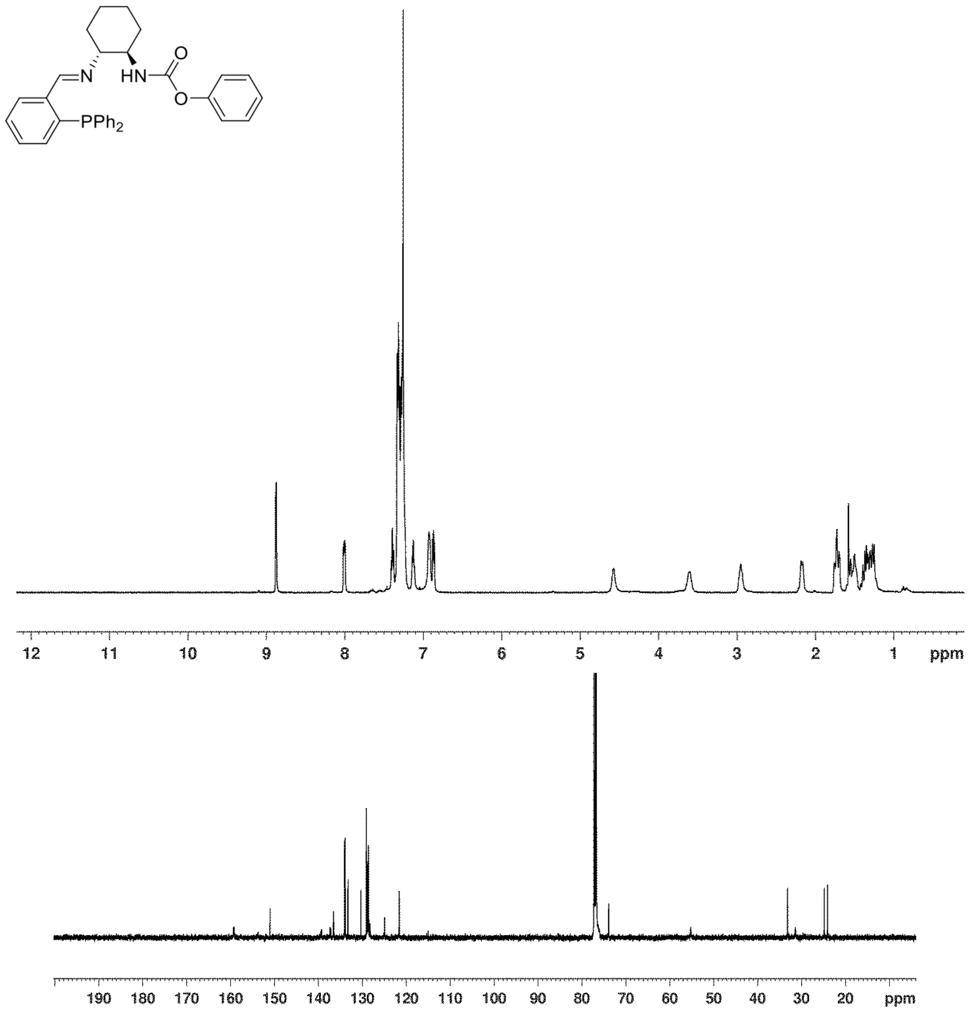

Figure S12. ^{1}H- and ^{13}C-NMR spectra of compound **D3**

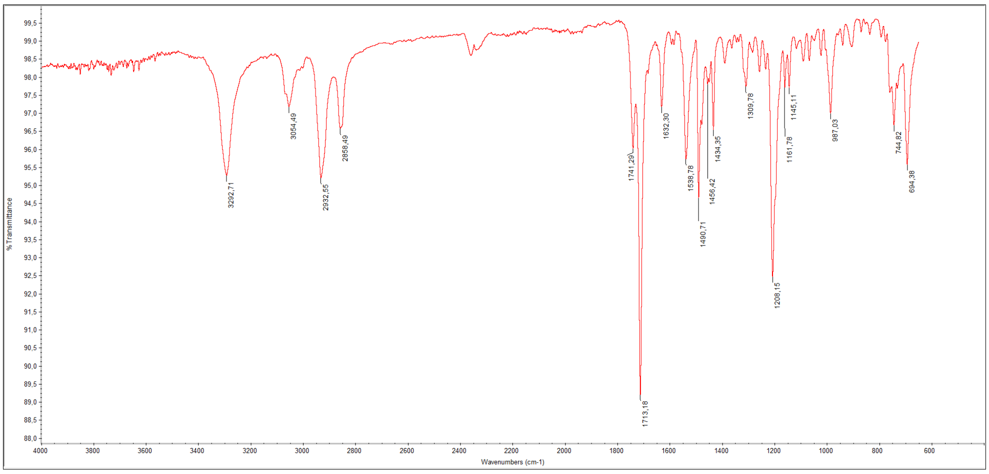

Figure S13. ATR-IR spectrum of compound **D3** (ATR corrected, neat)
